# Phage Orf Family Recombinases: Conservation of Activities and Involvement of the Central Channel in DNA Binding

**DOI:** 10.1371/journal.pone.0102454

**Published:** 2014-08-01

**Authors:** Fiona A. Curtis, Ali D. Malay, Alexander J. Trotter, Lindsay A. Wilson, Michael M. H. Barradell-Black, Laura Y. Bowers, Patricia Reed, Christopher R. T. Hillyar, Robert P. Yeo, John M. Sanderson, Jonathan G. Heddle, Gary J. Sharples

**Affiliations:** 1 School of Biological and Biomedical Sciences, Biophysical Sciences Institute, Durham University, Durham, United Kingdom; 2 Heddle Initiative Research Unit, RIKEN, Wako, Saitama, Japan; 3 Department of Chemistry, Biophysical Sciences Institute, Durham University, Durham, United Kingdom; University of Massachusetts, United States of America

## Abstract

Genetic and biochemical evidence suggests that *λ* Orf is a recombination mediator, promoting nucleation of either bacterial RecA or phage Red*β* recombinases onto single-stranded DNA (ssDNA) bound by SSB protein. We have identified a diverse family of Orf proteins that includes representatives implicated in DNA base flipping and those fused to an HNH endonuclease domain. To confirm a functional relationship with the Orf family, a distantly-related homolog, YbcN, from *Escherichia coli* cryptic prophage DLP12 was purified and characterized. As with its *λ* relative, YbcN showed a preference for binding ssDNA over duplex. Neither Orf nor YbcN displayed a significant preference for duplex DNA containing mismatches or 1-3 nucleotide bulges. YbcN also bound *E. coli* SSB, although unlike Orf, it failed to associate with an SSB mutant lacking the flexible C-terminal tail involved in coordinating heterologous protein-protein interactions. Residues conserved in the Orf family that flank the central cavity in the *λ* Orf crystal structure were targeted for mutagenesis to help determine the mode of DNA binding. Several of these mutant proteins showed significant defects in DNA binding consistent with the central aperture being important for substrate recognition. The widespread conservation of Orf-like proteins highlights the importance of targeting SSB coated ssDNA during lambdoid phage recombination.

## Introduction

Recombination in bacteriophages salvages genomes for packaging by restoring damaged or broken molecules via exonuclease processing and annealing. Illegitimate exchanges promote rapid evolution as new gene combinations or acquisitions can be generated during joint formation. In phage *λ* the Red system is responsible for exchanges at DNA ends [1]. Red*γ* protein serves to inhibit the *Escherichia coli* RecBCD exonuclease, ensuring that rolling circle replication can proceed [2], [3]. *λ* exonuclease (Red*α*) degrades the DNA duplex to generate 3′ ssDNA tails, which are bound by Red*β* protein, a strand annealing protein that searches for homologous ssDNA sequences [1], [4]. The combined action of Red*α* and Red*β* would be expected to favor splice-type recombinants, although there is evidence to indicate that annealing events occur regularly in the context of exposed ssDNA at a replication fork [5]–[7]. Thus, DNA synthesis primed by the 3′ annealed strand or by template switching can provide a means of generating intact genomes suitable for incorporation into capsids.

Phage *λ* encodes an ancillary recombinase called Orf, which was identified by its ability to complement the recombination deficiencies apparent in *recF*, *recO* and *recR* mutants of *E. coli* [8], [9]. The products of *recF*, *recO* and *recR* [10] function predominantly at ssDNA gaps and aid assembly of the primary bacterial recombinase, RecA, helping to overcome the inhibitory effects of ssDNA binding protein (SSB). Further *in vivo* studies revealed that the susceptibility of *recFOR* mutants to ultraviolet light could also be overcome by Orf [11]. Orf is a 17 kDa protein that behaves as a dimer in solution and interacts with *E. coli* SSB protein. It binds equally well to ssDNA, gapped duplex and 5′ and 3′ flap DNA and less well to fully duplex substrates [12]. The Orf crystal structure revealed an asymmetrical toroid with a central tunnel flanked by positively charged residues [12], [13]. Although the channel is too narrow to accommodate dsDNA, it could potentially encompass ssDNA. One side of the central cavity comprises a RAGNYA motif found in a wide range of DNA binding proteins [14]. DNA binding could also occur along a shallow groove across the protein surface since Orf bound equally well to a ssDNA flanked by duplexes, which would prohibit threading of ssDNA through the central hole [12]. Residues in the flanking C-terminal helices also contribute to stabilizing the association with DNA [13].

In this study we describe a family of phage and prophage Orf proteins exhibiting considerable sequence diversity but sharing common ancestry within a core domain. To substantiate their functional relatedness we purified one of the most dissimilar members, YbcN, from the *E. coli* cryptic prophage DLP12 and compared its properties with its counterpart from phage *λ*. YbcN was previously identified as a T:G mismatch and base-flipping enzyme [15], an activity that is not easily reconciled with a role in suppressing the genetic recombination and UV light repair defects of *recF, recO* and *recR* mutants.

YbcN behaved remarkably like *λ* Orf in its preference for binding ssDNA and in its association with *E. coli* SSB protein, supporting a functional relationship between the two phage proteins. However, some differences were noted in their interactions with DNA and SSB. YbcN bound much less well than Orf to ssDNA, although both proteins did show improved binding to bubble DNA structures. In addition, YbcN showed a specific interaction with the C-terminus of SSB, a feature not shared by *λ* Orf. Neither Orf nor YbcN exhibited an enhanced affinity for DNA containing a single G:G mismatch over fully duplex DNA, although Orf showed a slight preference for a T:G mispair. A number of mutants in *λ* Orf were generated to investigate the importance of the central cavity in DNA binding. Several of these mutants exhibited significant defects in DNA binding consistent with ssDNA passing through the interior of the Orf ring. These results, in combination with the ability of Orf to recognize bubble structures, support a clamp model for Orf assembly. The conservation of ssDNA and SSB binding activities in diverse members of the Orf family underscores the importance of facilitated loading of a partner recombinase for the successful initiation of phage recombination.

## Results

### The phage Orf family

A position-specific iterative BLAST search performed with the *λ* Orf protein identified homologs exclusively from phage genomes, predominantly Myoviridae, Siphoviridae and Podoviridae, or associated with prophage-like elements (Pfam05772 [16]). The initial BLASTP search uncovered multiple closely related sequences with an expectation value of less than 5×10^−5^. All of these proteins displayed greater than 30% identity with Orf. At the first PSI-BLAST iteration additional sequences with an expectation value of less than 4×10^−9^ were identified, including a highly diverged Orf-like protein (YbcN) from the DLP12 cryptic prophage of *E. coli* belonging to the PRK09741 conserved domain [17], currently classified as distinct from the PF05772 NinB (*λ* Orf) family. This gene matches almost exactly (97% identity) the *ybcN* (*orf151*) gene of phage 82 [18], yet shares only 16% overall identity with Orf. Further searches initiated with DLP12 YbcN recovered typical Orf proteins including those from Aa*φ*23, Stx2 and *λ* at the second PSI-BLAST iteration confirming the relationship. YbcN showed significant similarity to several prophage-associated proteins including *E. coli* O157:H7 (CP-933X; 93% identity), *Shigella sonnei* (81% identity), *Photorhabdus luminescens* (41% identity) and *Neisseria gonorrhoeae* (27% identity). The Phyre^2^ protein fold recognition tool was used to compare the predicted secondary structures of DLP12, *P. luminescens* and *N. gonorrhoeae* YbcN proteins with known three-dimensional structures in the structural classification of proteins (SCOP) database. In all three cases, the *λ* Orf fold was returned with >95% estimated precision. Representative Orf family proteins identified in this study varied in length from 124-181 residues; a list, including accession numbers, is available in Table S1.

A phylogram was prepared using the PAUP program to visualize similarity between these selected Orf family representatives (Figure S1). In this comparison there appear to be at least four discrete Orf groups occurring in phages from diverse species, although most belong to the *β* and *γ* proteobacteria. As might be expected, examples from closely related species tend to cluster together. For example, one of the groups contains highly related Orf proteins displaying 91-99% identity and is principally derived from *E. coli* O157:H7 or O84:H^-^ resident prophages (Figure S1, shaded in blue). However, this is not always the case as other clusters incorporate representatives from *E. coli* H phage HK620, *Actinobacillus actinomycetemcomitans* phage Aa*φ*23 and *Haemophilus influenzae* prophages. Indeed some orthologs, mainly from prophages residing in dissimilar bacterial species, do not fit unambiguously into any of the above groups. These discrepancies illustrate the difficulty in unraveling phage and prophage lineages due to high rates of horizontal gene transfer and mutation.

The *Listeria* proteins belong to the Orf family but are distantly related, sharing only 18% identity with *λ* Orf (Figure S1 and Table S1). Both *L. innocua* Orf-like proteins belong to yet another distinct conserved domain DUF968 (Pfam06147), which includes homologous polypeptides from *Staphylococcus, Lactobacillus* and *Enterococcus* phages and prophages. The N-terminal domain of these latter proteins share 23–24% identity with *λ* Orf consistent with a distant relationship with the Orf family. However, members of the DUF968 family possess an additional C-terminal extension, absent from the majority of Orf homologs, which incorporates a zinc finger HNH endonuclease motif [19]. These phages may therefore encode a novel single-strand specific deoxyribonuclease arising from fusion of an Orf DNA binding domain to an HNH nuclease domain. It is interesting to note that the DUF968 domain is found fused to a number of recombinases, including the strand annealing proteins, Erf and Rad52, and the Holliday junction resolvase, RusA [17]. The HNH region alone from this family is also found joined to the mismatch recognition factor, MutS.

### Orf family gene organization among lambdoid phages

**Figure 1:**
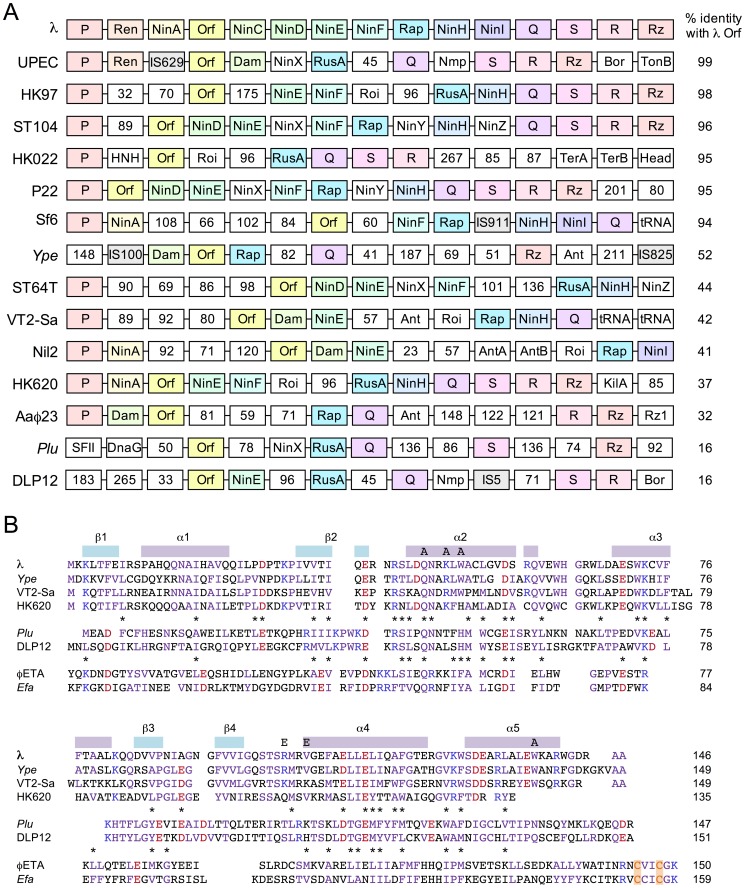
Conservation of genomic organization and protein architecture among selected members of the Orf family. (**A**) Conserved gene order of *orf* relative to the replication and lysis genes of lambdoid phages and prophages. Genes are shown as boxes and colored according to their order in the *λ* *ninR* region; in almost all cases they are transcribed from left to right. Orthologous genes are highlighted by name and color. Those that failed to match any of the *λ* genes in this region are shown as white boxes (the residue length of unannotated open-reading frames is indicated). Additional abbreviations refer to gene products that match the HNH nuclease domain family (HNH), DNA/RNA helicase superfamily II (SFII) or phage head proteins (head). The percentage identity to *λ* Orf is shown for each Orf homolog and listed in order of similarity. Many of the prophages are reservoirs for insertion sequences (shaded in grey); note that additional genes encoded by these elements are not shown. The putative 64-residue polypeptide between Q and S in *λ* was also omitted. (**B**) Conserved primary structure of *λ* Orf, DLP12 YbcN and ***φ***ETA Orf20 proteins. *λ* Orf is aligned with homologous representatives from *Y. pestis* (*Ype*), *E. coli* O157:H7 prophage Stx2-converting phage VT2-Sa and *E. coli* H phage HK620. DLP12 YbcN is aligned with a prophage homolog from *P. luminescens* (*Plu*). *Staphylococcus aureus* ***φ***ETA Orf20 is aligned with a homolog from *Enterococcus faecalis* (*Efa*). Conserved residues within each subset are highlighted in blue (basic), red (acidic) and lilac (others); those occurring in both Orf/YbcN and Orf/Orf20 alignments are marked with an asterisk. Secondary elements from the crystal structure of *λ* Orf [12] are indicated above the alignment, as are the site-directed mutants Q45A, K48A, W50A, R103E, V106E and W137A. A pair of cysteines from the HNH motif are highlighted in orange.

The *λ* *orf* gene lies in the *ninR* region between replication gene P and the Q antiterminator (Figure 1A). In addition to Orf, this section encodes two other genes associated with DNA metabolism: Rap (NinG), a DNA branch-specific endonuclease [20] and NinH, a dsDNA binding protein (G.J. Sharples, unpublished results). Where possible to determine based on homology of the surrounding genes, *orf* resides in a similar context in many phages and prophages, sandwiched between replication and lysis functions (Figure 1A). Significant shuffling of the genes in this region is evident with acquisition of unrelated genes, many of unknown function, and elimination of others. Despite these rearrangements, there are notable consistencies in gene order. For instance, preservation of synteny is particularly evident in the occurrence of the short *ninD*, *ninE* and *ninF* genes (Figure 1A). In every case *orf* lies upstream of *rap* or *rusA* genes, which specify structurally distinct Holliday junction DNA resolvases [21]. The genetic organization of the *orf*-like gene in DLP12 (*ybcN*) located just three genes 5' of *rusA* adds support to the notion that it is a genuine ortholog of *λ* Orf (Figure 1A).

The gene arrangement is radically different among the DUF968 (Pfam06147 [17]) Orf-like proteins from *Listeria* and *Staphylococcus* in keeping with phage sequences from different evolutionary origins. However, homologs of *dnaB*, *dnaC* or *ssb* are located close to the predicted *orf* gene in many of these sequences, linking these regions with functionality in DNA metabolism.

### Sequence similarity between *λ* Orf and DLP12 YbcN

Secondary structure predictions and modeling using Jpred and Phyre^2^ with DLP12 YbcN (and representatives of the DUF968 family) indicated a very similar overall architecture to that found with *λ* Orf (Figure S2). The main differences appear to be in the vicinity of *α*3 and *β*3 and in the C-terminal *α*-helix (Figure 1B). Any alterations in this first region could potentially affect dimerisation, although there are unlikely to be gross distortions in the overall fold. By examining Orf family sequence alignments alongside the crystal structure of *λ* Orf [12], functionally important residues in Orf and YbcN homologs were identified (Figure 1B). Nine hydrophobic residues from two discrete segments of Orf, namely *β*1-*α*1-*β*2 (comprising the RAGNYA motif [14]) and *α*3-*β*3-*β*4, contribute to the dimer interface. Four of these residues are conserved in YbcN homologs, while the remainder could be replaced by adjacent functionally equivalent amino acids (Figure 1B). Interestingly, the most highly conserved residues cluster around *α*-helix 2 and flank the positively charged channel that traverses the dimer [12]. Equivalents of *λ* Orf Arg41, Gln45, Asn46 and Arg48 are present in the majority of Orf sequences (Figure 1B, Figure S2 and data not shown). Arg41 from subunit B lies on the rim of the dimer cavity and could potentially contact the ssDNA backbone. In contrast, Arg41 in subunit A is hidden at the base of the cavity. Gln45 and Asn46 residues are symmetrically orientated and project from opposite sides towards the central channel. Asn46, in combination with Trp50 (which occurs as Trp or His in almost all Orf sequences), could interact with nucleotide bases if ssDNA can be induced to enter the ring interior. Finally, Arg48 is located at the rim of the aperture in each subunit, on opposite faces of the dimer, and could also make contacts with the C-terminal *α*-helix.

### Purification of DLP12 MBP-YbcN

To resolve whether members of the PRK09741 (YbcN) group are genuine functional equivalents of *λ* Orf, we set out to purify YbcN from *E. coli* DLP12 and validate its properties *in vitro*. Plasmids carrying the *ybcN* gene were constructed in order to overexpress wild-type and an N-His_6_ fusion of YbcN protein. High levels of expression were achieved, however, all of the YbcN recovered precipitated with the cellular debris upon lysis (data not shown). In an attempt to recover soluble protein we designed a construct that fuses the *E. coli* maltose binding protein (MBP) to the N-terminus of YbcN. Large quantities of soluble YbcN were recovered and the protein purified to near homogeneity by amylose and heparin chromatography. A GST-YbcN fusion was also purified at the same time using glutathione sepharose, heparin agarose and Q sepharose chromatography steps. MBP-YbcN and GST-YbcN proteins were analyzed in parallel with purified MBP-Orf and GST-Orf fusions from bacteriophage *λ* [13].

### YbcN binds preferentially to ssDNA

**Figure 2:**
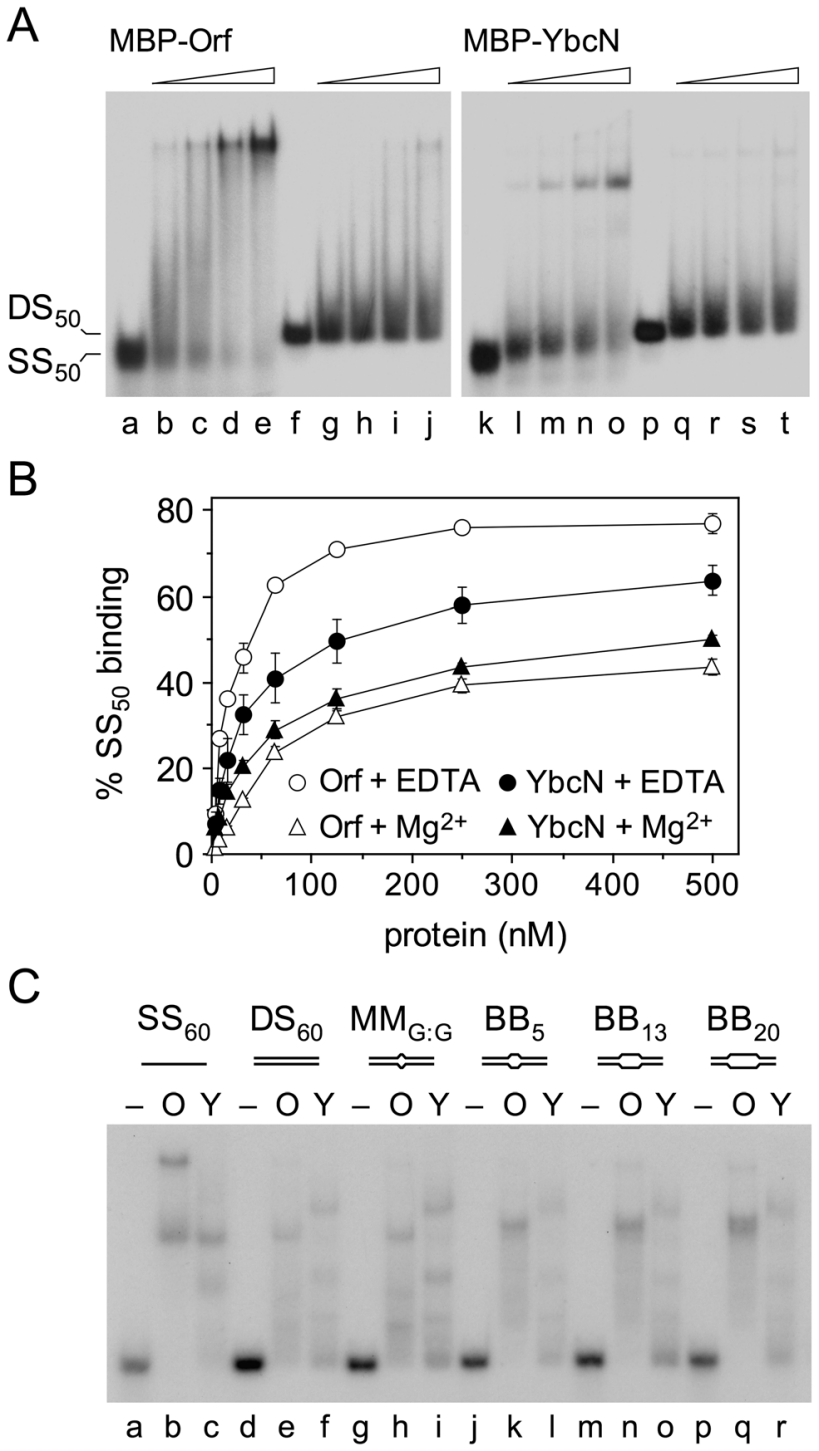
Comparison of MBP-YbcN and MBP-Orf binding to DNA. (**A**) Binding to ssDNA and dsDNA. Gel mobility shift assays contained 0, 62.5, 125, 250 and 500 nM MBP-Orf or MBP-YbcN proteins, 5 mM EDTA and either 0.3 nM of ^32^P-labelled 50 nt (SS_50_) ssDNA (lanes a-e and k-o) or 50 bp dsDNA (lanes f-j and p-t). (**B**) MBP-YbcN and MBP-Orf binding to ssDNA. Binding reactions contained 0.3 nM SS_50_ with 0, 7.81, 15.625, 31.25, 62.5, 125, 250 and 500 nM protein and either 5 mM EDTA or 1 mM MgCl_2_. Data are the mean and standard deviation of two independent experiments. (**C**) Binding to mismatch and bubble DNA. Gel mobility shift assays contained 250 nM MBP-Orf (O) or MBP-YbcN (Y) proteins, 5 mM EDTA and 0.15 nM of ^32^P-labelled 60 nt (SS_60_) ssDNA (lanes a-c), 60 bp (DS_60_) dsDNA (lanes d-f), 1 bp (MM_G:G_) mismatch (lanes g-i), 5 bp (BB_5_) bubble (lanes j-l), 13 bp (BB_13_) bubble (lanes m-o) and 20 bp (BB_20_) bubble (lanes p-r).

*λ* Orf protein favors binding to ssDNA over dsDNA in keeping with its predicted role in the initial steps of recombinational exchange [12]. To confirm that YbcN is functionally analogous to Orf, we investigated the ability of MBP-YbcN to bind short ssDNA (SS_50_) and dsDNA (DS_50_) substrates in an electrophoretic mobility shift assay carried out in the presence of EDTA to exclude metal ions. Similar to MBP-Orf (Figure 2A, lanes b-e), MBP-YbcN formed a single complex with the 50 nucleotide (nt) ssDNA substrate (Figure 2A, lanes l-o). In both cases smearing of the substrate is indicative of unstable protein-ssDNA complexes that tend to dissociate during electrophoresis. MBP-YbcN exhibited a lower affinity for ssDNA relative to MBP-Orf protein (Figure 2B). Replacing EDTA with 1 mM MgCl_2_, in both binding reaction and gel, significantly reduced binding of MBP-YbcN and MBP-Orf to SS_50_ (Figure 2B). MBP-Orf was much more sensitive than MBP-YbcN to inclusion of Mg^2+^ ions. No further reduction in ssDNA binding was observed when 10 mM MgCl_2_ was used (data not shown). The results are consistent with a counterion effect and protein-DNA interactions occurring through electrostatic contacts with the DNA backbone. Neither MBP-YbcN nor MBP-Orf bound well to 50 bp dsDNA, although both proteins did manage to form a stable complex with a small proportion of the substrate with smearing again consistent with a weak association (Figure 2A, lanes g-j and q-t).

To provide more quantitative data on the relative affinities of YbcN and Orf for ssDNA, we employed fluorescence anisotropy using a fluorescein-labeled SS_60_ substrate. This method relies on the fact that protein assembly onto DNA slows the spin of the fluorescently-labeled molecule in polarized light resulting in an increase in observed anisotropy (*r*_obs_). Studies are typically conducted with DNA substrates of <40 bp in length but larger substrates of 70 bp also yield reliable data [22]. As in the gel shift assays, MBP-YbcN bound much less well to ssDNA than MBP-Orf, with a 21-fold difference in their estimated dissociation constants (Table 1).

**Table 1:** MBP-YbcN and MBP-Orf mutant dissociation constants.

protein	SS_60_	BB_20_
YbcN	4602±275.1	1619±3.2
Orf wt	223±72.6	173±20.9
Q45A	272±20.4	264±15.2
K48A	622±0.7	551±6.4
W50A	3535±287.8	1606±418.2
R103E	–	–
V106E	522±12.4	540±73.2
W137A	628±99.9	285±1.9

Fluorescence anisotropy assays were performed in 100 mM Tris.HCl pH 7.0 with 10 nM fluorescein-labeled DNA. The apparent K_D_ (nM) was calculated for the protein as a dimer. Standard deviations of the duplicate data sets are shown.

### Orf and YbcN binding to mismatch, bubble and bent DNA

Since YbcN has been implicated in binding preferentially to DNA containing a T:G mismatch or abasic site [15], we compared the binding properties of MBP-YbcN and MBP-Orf on 60 bp DNA substrates with 1, 5, 13 and 20 nt unpaired regions, utilizing 60 nt ssDNA as a control. The larger bubble structures resemble structures more likely to be encountered during recombination rather than DNA mismatch recognition and repair. MBP-Orf formed two complexes with SS_60_ (Figure 2C, lane b), suggesting that the additional 10 nt permits assembly of two dimers of Orf compared to the 50 nt substrate (Figure 2A, lanes b-e). As noted before (Figure 2A), binding to dsDNA was less efficient and the complexes formed were prone to dissociation (Figure 2C, lane e). Inclusion of mismatches in the DNA affected the pattern of complexes formed, with BB_5_, BB_13_ and BB_20_ showing improved binding and formation of a more stable, distinct complex (Figure 2C, lanes k, n and q); the complex formed with these substrates is actually composed of two closely-migrating species, possibly indicating two alternative conformations of Orf bound to bubble DNA. Binding of MBP-Orf to the single G:G mismatch (Figure 2C, lane h) did not differ significantly from that seen with the fully paired substrate (Figure 2C, lane e).

MBP-YbcN formed two complexes on SS_60_ (Figure 2C, lane c) and three with DS_60_, although with a slightly altered mobility (Figure 2C, lane f). Surprisingly, as the length of unpaired DNA increased from 1-20 nt, there was no marked improvement in DNA binding or stability of complex formation (Figure 2C, lanes i, l, o and r). A 2.8-fold preference for BB_20_ over SS_60_ was apparent with MBP-YbcN in fluorescence anisotropy assays (Figure S3; Table 1). It is possible that the flanking duplexes in the bubble substrate help stabilize Orf, but not YbcN, complex formation by preventing protein dimers from sliding off the ends.

The absence of any preference for MM_G:G_ (a G:G mispair) relative to DS_60_ (compare Figure 2C, lanes f and i) could be because YbcN has a high degree of specificity for single T:G base pair mismatches [15]. To assess this possibility, an MM_T:G_ substrate with a T:G rather than a G:G mismatch was employed in fluorescence anisotropy assays. MBP-YbcN exhibited no significant differences in binding to MM_T:G_ or a fully duplex control (Figure S4A). MBP-Orf showed some preference for the DNA containing a T:G mispair suggesting some specificity for this type of replication error, although the data sets varied, consistent with a relatively poor association with duplex DNA (Figure S4A). Binding of MBP-YbcN and MBP-Orf was also tested on substrates containing centrally located insertions of 1, 2 or 3 adenines in one of the strands to generate bent DNA (Figure S4B). Adenine bulges of 1-3 nt are known to introduce a kink of 50-70° in duplex DNA [23], [24]. These substrates resemble mismatches by reducing the thermodynamic stability of the helix and inducing localized distortion of the DNA backbone. Neither protein showed any preference in binding these three substrates relative to DS_60_ (Figure S4B). Hence under the conditions employed here, neither YbcN nor Orf appear to favor bent DNA or duplexes where mispaired or bulged nucleotides might encourage the formation of extrahelical bases.

### YbcN binds SSB

**Figure 3:**
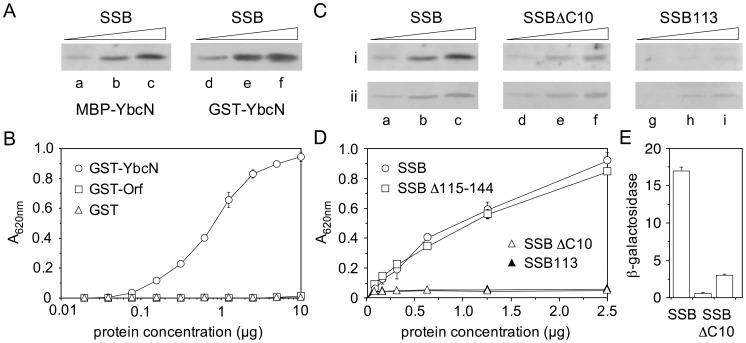
YbcN binding to SSB. (**A**) Interaction of YbcN with SSB in far western assays. SSB protein (0.5, 1.9, 3.8 µg) separated on 15% SDS-PAGE was blotted and probed with 20 µg MBP-YbcN (lanes a-c) or GST-YbcN (lanes d-f). Interactions were detected with antibodies specific for either the MBP or GST domains. (**B**) Interaction of GST-YbcN with SSB as detected by ELISA. SSB (0.5 µg) bound to Immulon-1 microtitre plates was exposed either to GST-YbcN, GST-Orf or GST proteins (0.0195-10 µg). Positive interactions were detected by addition of anti-GST antibody-HRP conjugate. Data are the mean and standard deviation of four independent experiments. (**C**) YbcN binding to SSBΔC10 and SSB113 proteins in far western assays. 0.5, 1.9, 3.8 µg SSB (lanes a-c), SSBΔC10 (lanes d-f) and SSB113 (lanes g-i) separated on 15% SDS-PAGE were blotted and probed with 20 µg MBP-YbcN. Interactions were detected with antibodies specific for MBP (row i). Row ii shows the efficiency of SSB, SSBΔC10 and SSB113 transfer onto blotted membranes as revealed by staining with amido black. Panel i (lanes a-c) is reproduced from (A) to facilitate comparisons with MBP-YbcN binding to wild-type SSB. (**D**) Interaction of GST-YbcN with SSB, SSBΔC10, SSB113 and SSBΔ115-144 as detected by ELISA. SSB wild-type and mutant proteins (0.5 µg) bound to microtitre plates were exposed to GST-YbcN (0.078-2.5 µg) and probed as in (B). Data are the mean and standard deviation of two independent experiments. (**E**) Yeast two-hybrid analysis of YbcN interactions with SSB and SSBΔC10. Experiments were performed using constructs fused to the GAL4 DNA-binding or activating domains. The β-galactosidase activities were the mean and standard deviation of three independent experiments. The vertical bars represent the β-galactosidase activity (Miller units) and values for YbcN-SSBΔC10 are given for pGBKT7-53 and pGADT7-T constructs in both orientations.

Previous studies [12], [13] revealed an interaction between *λ* Orf and *E. coli* SSB consistent with a role at an early stage in phage recombination. Far western blotting experiments were conducted as in these earlier studies to determine whether an association with SSB is conserved in YbcN. As with *λ* MBP-Orf, an interaction between MBP-YbcN and SSB bound to the membrane was detected with anti-MBP antibodies (Figure 3A, lanes a-c), suggesting that contacts between these proteins are preserved and are therefore functionally relevant. In fact, YbcN yielded a stronger signal than Orf [13] indicating that it has a higher affinity for SSB. No bands were detected using MBP alone to probe membrane-bound SSB protein showing that YbcN is necessary to detect binding to SSB. The association with SSB was further validated using a purified GST-YbcN fusion in far western blots probed with antibodies specific for the GST tag. The results mirrored those obtained with the MBP-tagged YbcN protein (Figure 3A, lanes d-f).

A GST-YbcN interaction with SSB, similar to a positive control containing GST captured on the plate, was also detected in an enzyme-linked immunosorbent assay (Figure 3B). GST-Orf failed to produce a signal suggesting that some of the SSB epitopes normally recognized by Orf may be occluded by the manner of SSB attachment to the surface of the plate. Addition of a 51-nt ssDNA at the outset or upon addition of GST-Orf or GST-YbcN did not significantly alter the results obtained (data not shown). In addition, YbcN-SSB complex formation was probed using the yeast two-hybrid system. A clear interaction was detected between YbcN and SSB (Figure 3E). *λ* Orf and *E. coli* SSB also associated in equivalent assays but much less strongly [13].

### The C-terminus of SSB is required for YbcN binding

Ten residues at the carboxy-terminus of *E. coli* SSB (168-PMDFDDDIPF-177) comprise a negatively-charged tail that functions as an assembly point for multiple proteins involved in DNA replication, recombination and repair [25]. *λ* Orf has previously been shown to bind SSB in far western assays in the presence or absence of this C-terminal region [13]. Similar experiments were carried out with MBP-YbcN using SSB113, which carries a single amino acid substitution in this region (P176S) and is known to confer a temperature-sensitive phenotype [26], and SSBΔC10, which lacks the last 10 residues entirely. Although SSB113 and SSBΔC10 proteins transferred less well to the membrane than wt SSB (Figure 3C, row ii, lanes a-i), it does appear that the MBP-YbcN interaction with the SSB C-terminal mutants is reduced relative to the wt, especially with SSB113 (Figure 3, row i, lanes a-i). Residual binding could potentially be due to traces of wt SSB in the SSB113 and SSBΔC10 samples as both mutant proteins were, by necessity, purified from strains carrying an intact *ssb* gene.

The possible reduced association between YbcN and the two mutant SSB proteins was investigated further using the enzyme-linked immunosorbent assay with SSB bound to the plate and anti-GST antibody used to detect any interaction with GST-YbcN (Figure 3D). In this assay, no binding between YbcN and SSBΔC10 or SSB113 was detected, whereas an interaction was readily observed with wt SSB. Another SSB deletion mutant, Δ115-144 [27], behaved like wt SSB, suggesting that this portion of SSB is dispensable for YbcN-SSB binding (Figure 3D). Anti-SSB antibodies were used to confirm that wt SSB, SSB113, SSBΔC10 and SSBΔ115-144 proteins all bound similarly well to the microtiter plate (data not shown). In addition, yeast two-hybrid analysis with the SSBΔC10 mutant showed a significant reduction in binding to YbcN (Figure 3E). Taken together, the results are consistent with the last 10 residues of SSB being necessary for a stable YbcN interaction. This contrasts with experiments showing that *λ* Orf retains the capacity to associate with SSBΔC10 and SSB113 mutants [13].

### Secondary and quaternary structure of MBP-YbcN

CD spectroscopy was used to provide information on the secondary structure and folding of MBP-YbcN and GST-YbcN proteins. MBP-YbcN yielded a typical spectrum for a globular protein and appears to be folded correctly (Figure S5A). The predicted proportion of helices, sheet, turn and coil are similar to those observed with MBP-Orf, in keeping with a similar overall fold (Table S2). In contrast, GST-YbcN appears rather more disordered (Figure S5A) and secondary structure predictions do not differ significantly from GST protein alone (Table S2). However, the protein does appear to be functional in binding to SSB (Figure 3) and ssDNA (Figure S5A, lanes j-l).

Size exclusion chromatography was employed to investigate the oligomeric state of MBP-YbcN and GST-YbcN proteins. MBP-YbcN eluted with a relative molecular mass of 113 kDa in agreement with the 120 kDa predicted for a homodimer (Figure S5B). The dimeric species is relatively stable and can be detected as an ∼170 kDa species in SDS-polyacrylamide gels (Figure S5B). Hence both YbcN and *λ* Orf [13] exist as a homodimer in solution. Gel filtration analysis of GST-YbcN, however, identified three major peaks that could correspond to tetramer, octamer and a larger multimeric species (Figure S5B). Stable tetrameric species of 140-150 kDa (a monomer of GST-YbcN is expected to yield a 44.5 kDa protein) were observed in SDS polyacrylamide gels (Figure S5B). Since GST is known to dimerize [28], higher molecular mass complexes consisting of dimer-dimer combinations are not unexpected.

### MBP-Orf substitution mutants flanking the central cavity

**Figure 4:**
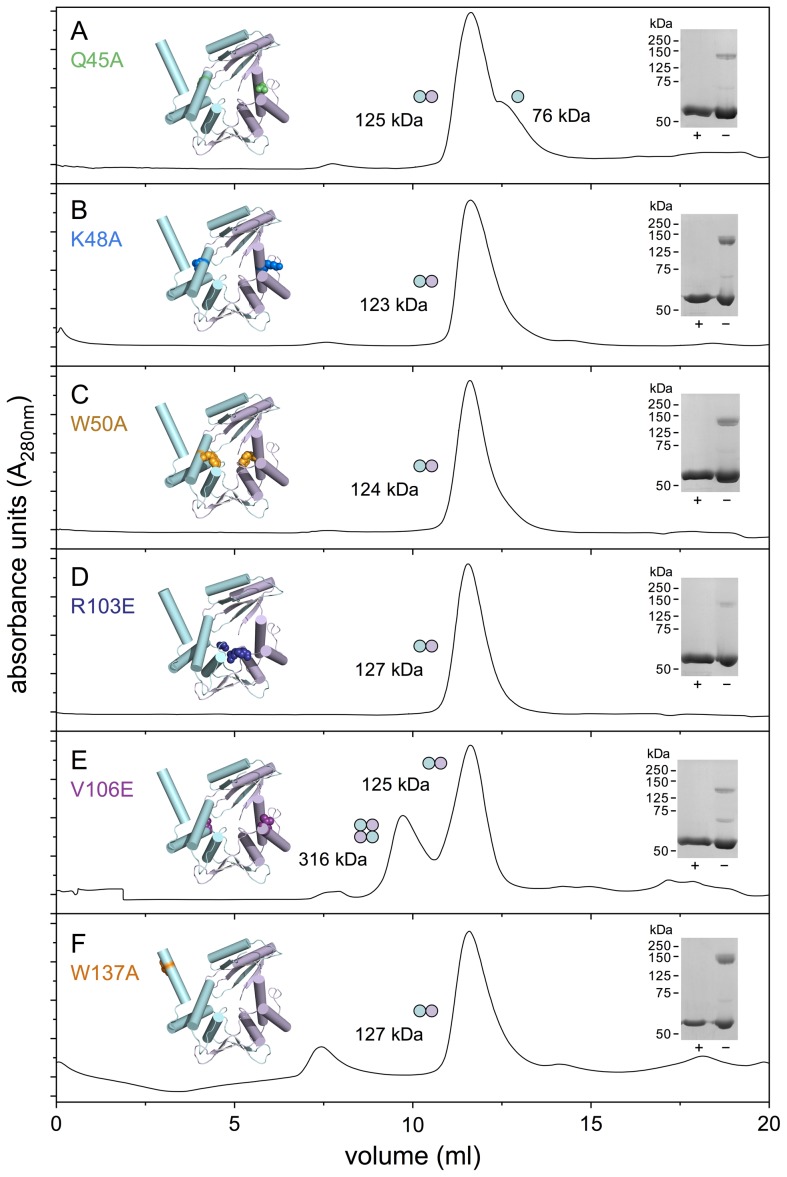
Size-exclusion chromatography of MBP-Orf mutant proteins. Proteins (1 mg/ml) were applied to a 24 ml Superose 12 HR 10/30 column. In each panel, the crystal structure of Orf protein shows the location of the relevant substitution mutant. Chain A is colored cyan and Chain B, lilac; the projecting C-terminal helix is absent from the latter. Predicted molecular weights for MBP-Orf are 59.6 kDa (monomer) and 119.2 kDa (dimer). Oligomeric states are depicted with a circle representing a single subunit and placed adjacent to the corresponding peak. Boiled (+) and unboiled (–) samples of each purified protein were separated on 12.5% SDS-PAGE and stained with Coomassie blue.

A number of site-directed mutants were constructed in *λ* Orf to help identify residues critical for interaction with ssDNA (Figure 4). Alanine substitution mutants were introduced in an aromatic residue projecting into the central cavity (W50A), as well as two nearby polar residues at the channel entrance (Q45A, K48A). These residues match several that are conserved within the Orf family (Figure 1B and Figure S2). More radical glutamic acid substitutions (R103E, V106E) were generated further from the cavity entrance that could also potentially participate in stabilizing contacts with DNA (Figure 4). The latter two mutations were also chosen as they lie in a motif (102-SRMRVGE-108) that resembles a region at the subunit-subunit interface in the *E. coli* RecA filament (25-SIMRLGE-31) [29], [30] and could potentially mimic a RecA monomer to nucleate polymerization as a recombination mediator. A related motif has been identified in BRCA2 that promotes assembly of Rad51, the human RecA ortholog, at ssDNA gaps [29], [31]. An additional alanine substitution was introduced in the C-terminal helix (W137A) as it was anticipated that it would behave similarly to mutants obtained previously in this region [13], which are known to impair DNA binding to linear ssDNA and dsDNA. All mutant proteins were purified as fusions with maltose binding protein to improve Orf solubility, simplify the recovery of milligram quantities of pure protein and facilitate far-western assays for protein-protein interactions.

### Quaternary and secondary structure of MBP-Orf mutants and effect on SSB binding

To confirm that the six mutations did not substantially affect dimerization or folding, size exclusion chromatography, static light scattering experiments and circular dichroism (CD) spectroscopy were performed. The quaternary structure of each mutant was assessed by size exclusion chromatography. The results reveal that the mutant proteins tend to elute as a single major peak of 123-127 kDa (Figure 4) in keeping with the 120 kDa predicted for an MBP-Orf homodimer. Dimeric species of 135-140 kDa resistant to denaturation were also observed on SDS-PAGE when purified samples were separated in the absence of a reducing agent and without boiling (Figure 4). The Q45A mutant showed an additional peak in gel filtration that corresponded to the size of a monomer (Figure 4A) that may indicate that this residue normally contributes to dimerization, although this may not be a direct effect since it is located far from the subunit interface. Gel filtration analysis on V106E revealed two major peaks with the larger species consistent with formation of a pair of Orf dimers (Figure 4E). However, when samples were reexamined at pH 7, instead of pH 8, a single dimeric peak at 125 kDa was observed at both 0.5 mg/ml or 1 mg/ml protein (data not shown). Thus electrostatic interactions may be important in the formation of multimeric complexes in this mutant.

**Figure 5:**
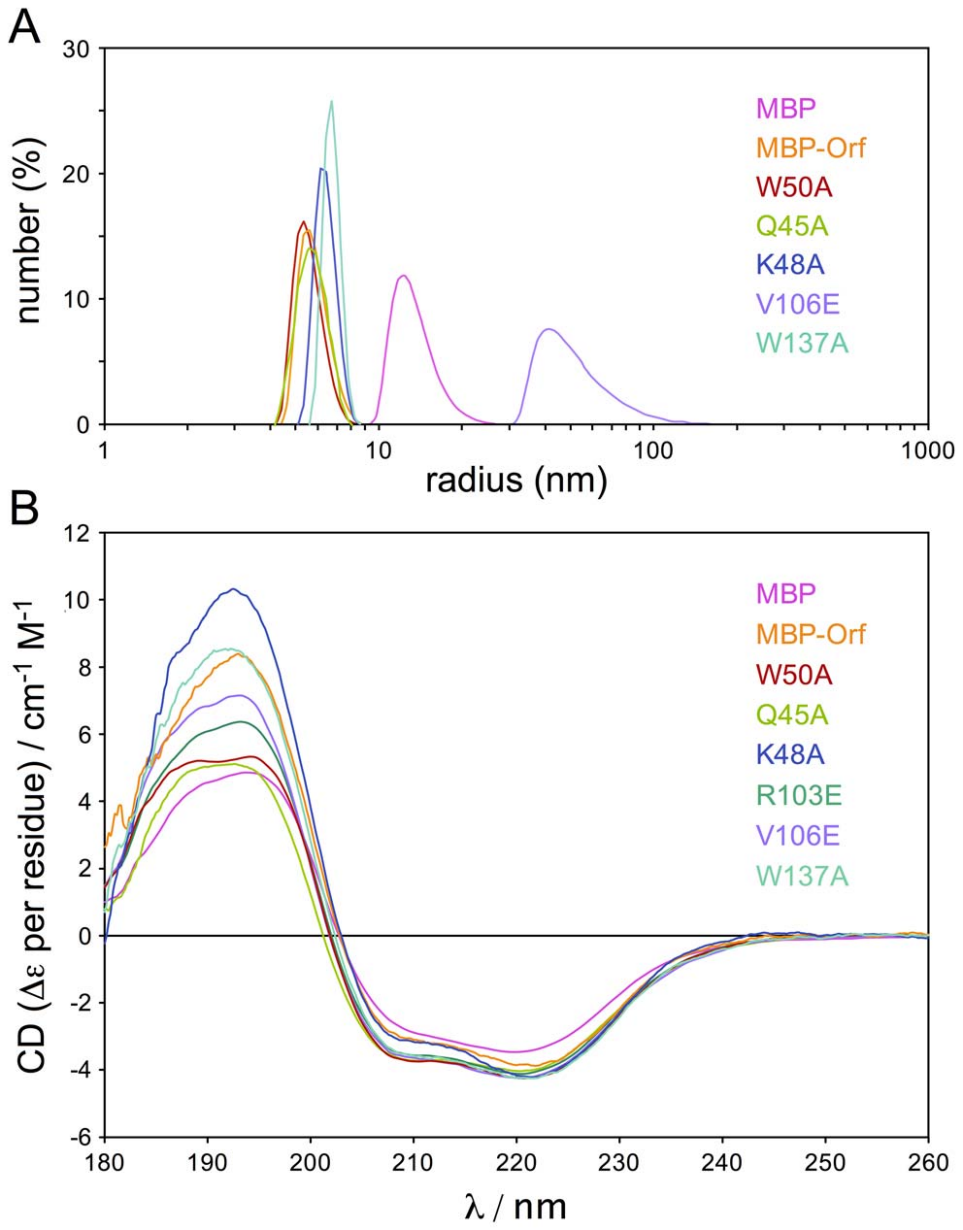
CD spectra and SLS analysis of MBP-Orf mutant proteins. (**A**) SLS analysis of selected MBP-Orf mutant proteins. Proteins at 0.2 mg/ml in 100 mM Tris-HCl pH7 at 25°C were analyzed in a Zetasizer µV. (**B**) CD analysis of MBP-Orf mutant proteins. CD spectra (180-260 nm) were obtained for MBP-Orf proteins in ultrapure water at 20°C and analysis performed using CDSSTR, Contin/LL and Selcon3 programs from the CDPro suite [54].

Each of the mutants was also examined by static light scattering, which determines the particle size in solution and provides an estimate of the oligomeric status of a protein sample. MBP-Orf yielded a 6.1 nm particle radius and similar sizes of 5.2-5.9 nm were obtained with Q45A, K48A, W50A, R103E and W137A (Figure 5A and Table S3). These correspond to an average molecular mass of 188 kDa, about 1.6 times that of an MBP-Orf dimer, although estimates are based on the assumption that a particle is spherical. The smallest dimension of 5.2 nm was obtained with R103E potentially indicating some conformational change affecting particle shape (Table S3). The V106E mutant differed from the other mutants (Figure 5A) in producing a large particle size with a radius of 114.9 nm (Table S3). It also showed a high percentage polydispersity of 55.8% suggestive of protein aggregation. Under these conditions, MBP also produced an apparent multimer of 17.9 nm (Table S3), which is in accord with previous gel filtration data indicating it exists as a large protein complex [13].

The mutant MBP-Orf proteins were also studied by CD spectroscopy to assess secondary structure and folding properties. All six mutants appear to be folded correctly (Figure 5B) and secondary structure predictions suggest that the R103E, V106E and W137A do not differ significantly from wt MBP-Orf (Table S2). The three mutants clustered around the central channel on *α*2 of the Orf dimer, Q45A, K48A and W50A, do however show differences in the proportion of predicted *α*-helix, *β*-sheet and turn (Table S2). K48A showed the most dissimilar CD spectrum in the far-UV region (Figure 5B) with a significantly increased percentage of *α*-helix and a corresponding decrease in both *β*-sheet and turn (Table S2). Hence, although these three proteins are folded and dimerize normally, the alanine substitutions may affect the local conformation of *α*2 and adjacent secondary structures.

Taken together the data do not reveal any substantial changes in mutant protein folding or homodimer formation, although subtle changes in secondary structure were observed with Q45A, K48A and W50A. V106E has a propensity to form multimers as noted in gel filtration and static light scattering experiments. In the case of the latter mutant it is possible that the presence of an additional negative charge on the surface encourages protein-protein interactions between Orf dimers that are generally positively charged over their protein surface.

Far-western blotting experiments were performed to determine whether any of the Orf mutants affected the association with *E. coli* SSB. None of the mutants showed any reduction in interaction with SSB (Figure S6), suggesting that contacts between the two proteins must be located elsewhere.

### Effect of MBP-Orf mutations on DNA binding

**Figure 6:**
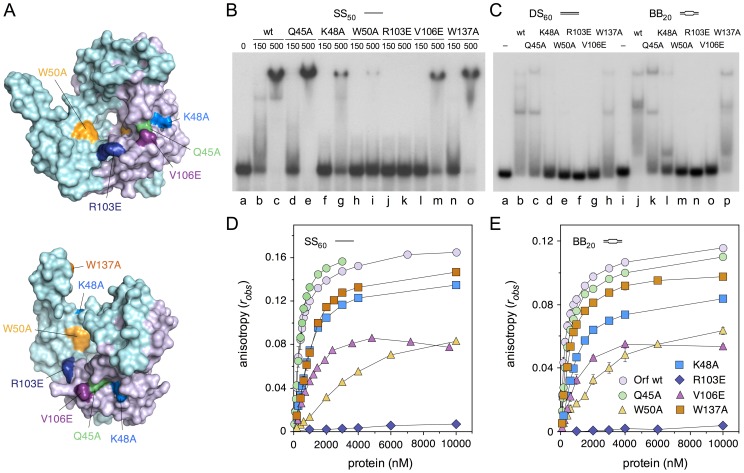
DNA binding by MBP-Orf mutant proteins. (**A**) Crystal structure of Orf protein (1PC6) highlighting the location of site-directed mutations. Chain A is colored cyan and Chain B, lilac. The projecting C-terminal helix is absent from the latter. (**B**) MBP-Orf mutant binding to ssDNA. Gel mobility shift assay contained 0.3 nM ^32^P-labelled ssDNA (SS_50_) and protein at 150 and 500 nM as indicated. Samples were separated on 4% polyacrylamide gel electrophoresis and visualized by autoradiography. (**C**) MBP-Orf mutant binding to double-stranded and bubble DNA. The gel shift assay contained 0.15 nM ^32^P-labelled dsDNA (DS_60_; lanes a-h) or bubble DNA (BB_20_; lanes l-p) and 250 nM MBP-Orf protein. Samples were separated by 4% polyacrylamide gel electrophoresis and visualized by autoradiography. (**D** and **E**) MBP-Orf mutant binding to 10 nM fluorescein-labeled ssDNA (D) and BB_20_ DNA (E) as determined by fluorescence anisotropy. Binding isotherms are colored as in (A). Data are the mean and standard deviation of two independent experiments.

The impact of the six MBP-Orf substitution mutants (Figure 6A) on ssDNA and bubble DNA binding was examined in electrophoretic mobility shift and fluorescence anisotropy assays. Binding to a 50 nt radiolabeled linear ssDNA was examined first (Figure 6B) in the absence of metal ions. MBP-Orf wt forms a single major complex with the ssDNA (Figure 6B, lanes a-b), with smearing consistent with an unstable interaction with the substrate as noted previously [13]. The Q45A mutant showed a minor defect in binding as evidenced by the increased presence of unbound substrate (Figure 6B, lane d) relative to the wt. W137A also showed a slight reduction in ssDNA binding, similar to that seen with a W141F mutant [13] and consistent with the *α*5 helix contributing to DNA binding stability. More severe defects in ssDNA binding were evident with K48A and V106E, while W50A bound only weakly (Figure 6B). No DNA binding was detected with the R103E mutant (Figure 6B, lanes j-k).

Gel shift experiments were also performed on dsDNA (DS_60_) and 20 nt bubble (BB_20_) substrates (Figure 6C). The results generally mirrored those with SS_50_ (Figure 6B), although they highlighted more severe DNA binding defects with several of the mutants. While wt MBP-Orf binds relatively poorly to DS_60_ (Figure 6C, lane b), K48A, W50A, R103E and V106E displayed little or no association with this substrate (Figure 6C, lanes d-g). Similarly poor binding was observed with the bubble DNA, although K48A did bind a small proportion of BB_20_ but only as a much faster-migrating complex (Figure 6C, lane l). Q45A and W137A showed less extreme defects on DS_60_ (Figure 6C, lanes c and h). However, the stability of complexes detected on BB_20_ was reduced slightly with W137A (Figure 6C, lane p) and significantly with Q45A (Figure 6C, lane k) compared to the wt. Interestingly, Q45A showed an inability to form the two closely-migrating complexes seen with the wt and W137A on BB_20_ (Figure 6C, compare lanes j, k and p).

Fluorescence anisotropy was again used to give quantitative information on binding to SS_60_ and BB_20_ substrates. Binding isotherms are presented (Figure 6D-E) alongside dissociation constants derived from the data (Table 1). The results match well those obtained in gel shift assays (Figure 6C) with gradually decreasing DNA binding activity in the order wt, Q45A, W137A, K48A, V106E, W50A and R103E. However, differences were noted within and between the two substrates that were particularly evident in the estimated K_D_ for each mutant protein (Table 1). In all cases, Q45A showed only a slight defect in DNA binding relative to wt. Similarly, R103E was the most severely deficient in DNA binding, followed by W50A. The remaining three mutants varied in order depending on the substrate. V106E bound better than K48A and W137A to SS_60_, whereas W137A bound more tightly than V106E and K48A to BB_20_ (Table 1). W50A also showed a preference for binding to BB_20_ over SS_60_. The observed anisotropy gradually decreased in the V106E mutant at concentrations above 4 µM with both SS_60_ and BB_20_ (Figure 6D-E). This could be due to a contaminant nuclease destroying the substrate, but it is more likely that V106E aggregates as the amount of protein increases resulting in a reduction in protein available for DNA binding. This conclusion fits with the data in Figures 4E and 5A showing that V106E has a tendency to form higher molecular mass complexes.

## Discussion

Phage *λ* Orf substitutes for the *E. coli* RecFOR proteins in genetic exchange reactions, possibly by directing assembly of either bacterial RecA or phage *β* recombinases onto ssDNA coated with SSB [8], [11]. Consistent with this role, purified Orf interacts with both *E. coli* SSB and ssDNA substrates [12], [13]. In this study we identified a family of Orf recombinases from diverse lambdoid phages sharing similarity in primary sequence, predicted secondary structure and genomic context within a conserved module located between replication and lysis genes. The authenticity of the proposed family relationship was investigated by studying the biochemical properties of YbcN, one of the most distantly-related members of the Orf family. YbcN is encoded by the *E. coli* cryptic lambdoid prophage DLP12, is 97% identical to a protein from phage 82 [18] and has previously been shown to bind preferentially to abasic sites and T:G mismatches, with the capacity to flip mispaired bases out of the double helix [15]. However, an involvement of YbcN in mismatch recognition does not seemingly fit with an activity responsible for suppressing the recombination and UV light repair deficiencies of *recFOR* mutants. The specific DNA binding properties of YbcN were therefore examined in direct comparison with *λ* Orf.

YbcN shared with Orf a preference for binding to short 50 or 60 nt ssDNA substrates rather than duplexes of the same length and sequence. However, YbcN bound much less well to ssDNA than Orf in the absence of metal ions, whereas their binding profiles were more similar in the presence of Mg^2+^ ions (conditions more closely resembling those found in the cell). No difference in binding was detected between a fully complementary dsDNA and one containing a single T:G or G:G mismatch with YbcN protein, although Orf did show a slight preference for the T:G mispair. YbcN showed no improved binding to related substrates containing a bubble of 5, 13 or 20 nt in gel shift assays, although a slight preference for 20 nt bubble DNA was evident in fluorescence anisotropy assays. In contrast, Orf bound much more tightly to these bubble DNA structures in keeping with its ability to load on ssDNA. It is possible that YbcN has a larger footprint than Orf and therefore requires a longer stretch of ssDNA to assemble in a stable complex. Alternatively, it may be that a free end is necessary for loading of YbcN, and that the flanking duplexes in these bubble substrates prevented assembly. The DNA binding properties of YbcN, and the observation that it forms homodimers, are in accord with a recombinase that is homologous to *λ* Orf and are consistent with a role at the early stages of recombination, binding to ssDNA exposed by phage exonuclease or targeting replication forks or bubbles in the template.

A high-throughput screen of *E. coli* protein-protein interactions had previously indicated an association between YbcN and SSB [32]. Far western blotting, ELISA and yeast two-hybrid experiments confirmed this interaction, with YbcN showing a higher binding affinity for its bacterial partner than Orf. These results confirm that a distantly-related Orf family member has the capacity to bind bacterial SSB and supports the notion that they form a conserved, functional complex. Multiple members of the replication, recombination and repair apparatus have been shown to target the negatively-charged C-terminus of SSB [25], presumably to promote delivery at sites requiring their action. Previous studies had revealed that the terminal 10 residues of SSB were dispensable for *λ* Orf binding [13]. In contrast, YbcN showed significantly reduced binding to SSB mutant proteins lacking this region (ΔC10) or carrying an SSB113 mutation, known to disrupt interactions with partner proteins [33], [34]. Some residual binding of YbcN to SSB was detected by far western blotting and yeast two-hybrid assays, suggesting there may be other portions of the flexible C-terminus of SSB (residues 113-177) or elsewhere that are more important for the Orf-SSB association. It may be that the region on SSB targeted by Orf contributes to remodeling of the SSB-ssDNA complex that allows loading of recombinase onto the exposed ssDNA. It is noteworthy that while both YbcN and Orf interact specifically with *E. coli* SSB, they appear to do so by recognizing disparate features on their bacterial partner.

Although there are differences in YbcN and Orf binding to DNA, their preference for single-stranded DNA does support the prediction that they belong to a conserved family with a comparable role in genetic recombination. It is difficult to reconcile any specificity for abasic sites and mismatches [15] with an activity that suppresses defects associated with mutation of the *recF*, *recO* or *recR* genes, unless the Orf domain has been co-opted to generate a base-flipping enzyme in YbcN. Neither Orf nor YbcN share any homology with enzymes that might incise DNA at a mismatch (e.g. a DNA glycosylase or apurinic/apyrimidinic endonuclease) or to modify any flipped out bases (e.g. a methyltransferase). Moreover, under the assay conditions used here, no specificity for G:G or T:G mismatches was detected with YbcN protein. One possibility is that Orf and YbcN can in certain circumstances capture and stabilize exposed DNA bases as part of their normal function in recognizing ssDNA for assembly of partner recombinases. Such an activity may ensure that a sufficient amount of ssDNA is released from SSB-ssDNA complexes to permit nucleation of a partner recombinase. RecOR are known to accelerate nucleation of RecA on SSB-coated ssDNA, apparently by trapping transient states where an SSB tetramer slides or unwraps from the DNA strand [35]. Recognition of SSB by YbcN and Orf, as with RecO [36], [37], would allow targeting of appropriate ssDNA substrates even if the protein-protein interactions do not directly contribute to SSB dissociation [36].

The prophage encoded YbcN could contribute directly to homologous recombination reactions in *E. coli*, although experiments with *rusA* [18], which lies downstream of *ybcN* in DLP12, suggest that insertion mutations that supply an active promoter upstream of this operon are needed for adequate gene expression at this locus. However, the presence of homology with other lambdoid phages in this region does mean that *ybcN* can serve as a repository of alternative phage genetic material that can be picked up by incoming phage, thus contributing to the ongoing evolution of mosaic genomes [38]. The presence of homologs of the defective prophage DLP12 *ybcN* gene in functional phage genomes, including 82 and ***φ***80, suggests that it does specify an active Orf-like protein.

The crystal structure of Orf [12] suggested two possible ssDNA binding modes, either negotiating the narrow (8 Å) central channel or bound within a shallow groove running perpendicular to this cavity. Binding to a gapped duplex substrate, and the bubble DNA substrates used in this study, implied that the latter interaction was the genuine one [13]. However, the experiments did not exclude the possibility that the dimeric ring opens to permit ssDNA access. Electrostatic potential calculations performed on the Orf crystal structure support this possibility as the interior of the toroid is highly positively charged [12]. Furthermore, several of the most highly conserved residues in the Orf family line the walls of this cavity. The identification of a new DNA binding fold in the N-terminal portion of the Orf structure also fits with this idea. This domain, termed RAGNYA [14], comprises two *α*-helices (*α*1 from each monomer in Orf) and four *β*-strands (*β*1 and *β*2 from each Orf subunit) with the predicted DNA binding surface across the face of the sheet region and within the central cavity. It may be that additional contacts involve the C-terminal helix, which protrudes from either side of the dimeric ring (Figure 4) and has previously been shown to participate in DNA binding [13].

Mutants in Orf at several conserved sites within or at the entrance to the central channel, alongside a substitution in the C-terminal helix, were studied to help clarify their contribution to ssDNA recognition. The W50A mutant protein exhibited a significant defect in DNA binding providing the first clear evidence that the central cavity is important for stabilizing contacts with ssDNA. Trp50 projects from both sides into the space within the dimer interior and could stabilize binding to nucleotide bases as they enter the channel. Lys48 is located nearby on the cavity rim and a K48A mutation also conferred a significant defect in DNA binding. This residue may make contact with *α*-helix 5 and either directly or indirectly affect ssDNA that may traverse the groove between this helix and the main body of the dimer, potentially allowing ssDNA to wrap around the toroid. Surprisingly, the Q45A mutant displayed only a minor defect in DNA binding activity compared to the wt, despite being highly conserved among the Orf family. It may play a more subtle role as the formation of protein:DNA complexes in gel shift assays showed some variation from the wt on both bubble and dsDNA structures.

Mutation of Trp137 to alanine resulted in a modest reduction in DNA binding, comparable to other substitution mutants in the Orf C-terminal helix (R132A, R140A and W141F) [13]. This entire helix is flexible and could adopt a variety of conformations to clamp onto DNA or associate with the RAGNYA domain as suggested previously [13]. The two remaining mutants, R130E and V106E, are located close to the cavity entrance. V106E showed a significant defect in DNA binding, while R103E was completely defective in binding any of the DNA substrates tested. The V106E mutant had a tendency to aggregate and thus it may serve in a structural role, stabilizing intrasubunit interactions between helices *α*2 and *α*4. Addition of a negatively-charged residue close to the rim of the channel could also disrupt electrostatic interactions between Orf and the DNA backbone. The R103E mutant could behave in a similar fashion as it lies at each end of the central channel and could be critical for stabilizing contacts with ssDNA. Arg103 in one of the Orf monomers forms a salt bridge with both Asp68 and Glu70 from the other subunit, however, the R103E mutant does appear to fold and dimerize normally suggesting that it does not significantly disrupt the dimer interface. If the Orf protein can open as a clamp, the R103E substitution could potentially affect opening and closing of the ring preventing its association with DNA. Arg103 and Val106 lie in a short motif that resembles part of the subunit interface in RecA [29], [30] and this region protrudes on both sides of the Orf cavity, ideally situated to aid nucleation of RecA onto ssDNA. However, as yet, we have been unable to confirm a physical interaction between Orf and RecA.

The assembly of Orf on ssDNA *in vivo* could facilitate loading of either the bacterial RecA strand exchange protein or phage *β* strand annealing protein to stimulate recombination [1]. Assuming that Orf behaves similarly to *recF*, *recO* and *recR*, the genes it substitutes for *in vivo* [8], [11], it could help nucleate RecA at replication forks, D-loops (similar to bubble structures) or at gaps resected in duplex DNA. This scenario would be analogous to bacteriophage T4, whereby the UvsY mediator promotes assembly of the UvsX recombinase (a RecA homolog) onto ssDNA, overcoming the block presented by the phage Gp32 ssDNA binding protein [39]. RecFOR are known to help RecA filaments assemble at dsDNA-ssDNA junctions [40]–[42], destabilizing SSB-ssDNA complexes that normally prevent access to the template [35]. The fact that RecFOR promote RecA filament assembly at gaps with either DNA or RNA at the 5′ terminus suggests that the stalled replication fork may well be a target for their mediator function [43]. Facilitated loading of RecA in this context could sanction lesion bypass and replication restart downstream of the block. Restoration pathways that depend on fork regression have been suggested [44], [45] and there is evidence that RecFOR play an important role [46] that can function independently of RuvAB-mediated Holliday junction processing [47]. If Orf could stimulate a similar recovery pathway, even by simply safeguarding the fork, this might explain why it can also partially substitute for the activities of RuvABC in Holliday junction branch migration and resolution [11].

Orf suppresses the recombination and DNA repair defects of *recFOR* mutants even in the absence of Red*α* *β* [8], [11]. However, recent experiments examining recombinational exchanges between partially homologous partners on phages and prophages, suggest that Orf is required primarily for exchanges promoted by the Red system rather than by RecA [38]. It is possible that RecFOR normally prevail over Orf in mediating the loading of RecA onto ssDNA bound by SSB, whereas the bacterial activities are less active in situations involving the Red system (e.g. at DNA ends). Depending on the context and availability of recombination enzymes, it may be that Orf aids nucleation of either phage Red*β* or bacterial RecA to promote recombinational exchange. Both RecA (strand invasion) and Red (annealing) pathways [48] can promote the recovery of viable phage genomes, although the latter may lead to more rearrangements at sites of limited sequence homology, thus fostering phage genetic diversity and mosaicism [38].

## Materials and Methods

### Sequence analysis

Proteins related to Orf were identified using the TBLASTN and PSI-BLAST algorithms [49] on the non-redundant database at the National Center for Biotechnology Information (NIH, Bethesda, MD). An inclusion threshold of 0.05 was applied in iterative searches to restrict those matches selected in the position-specific weight matrix (PSSM). Sequences were aligned using ClustalW with a Gap Creation Penalty of 10 and Gap Extension Penalty of 1 [50]. Phylogenetic relationships were estimated by the neighborhood-joining method incorporated within the PAUP package [51]. The Jnet protein structure prediction tool on the Jpred server was used to estimate secondary structure features of *E. coli* DLP12 YbcN and *S. aureus* *φ*ETA Orf20 [52]. The Phyre^2^ protein fold recognition server is an improved version of 3D-PSSM [53].

### MBP and GST fusion constructs

The *orf*-like gene (*orf151* or *ybcN*) from *E. coli* prophage DLP12 [18] was amplified from AB1157 genomic DNA using Pfx DNA polymerase and oligonucleotides 5′-CGGAGGGAATTCATGAACCTCTCAC-3′ and 5′-GACGATTTGGATCCCTGTAGATGTG-3′. The PCR product was digested with EcoRI and BamHI (underlined) and inserted into pMALc2 to give pLB101. This construct expresses *E. coli* MBP (maltose binding protein) fused to the N-terminus of YbcN. An N-terminal GST (glutathione S-transferase) fusion was constructed by transferring the insert from pLB101, using EcoRI and SalI sites, into pGEX-6P-1 (GE Healthcare) to generate pLB108 (GST-YbcN).

The QuickChange (Stratagene) site-directed mutagenesis method was used to generate Q45A (pFC256), K48A (pFC262), W50A (pFC259), R103E (pFC265), V106E (pFC267) and W137A (pEH101) mutant derivatives in pMALc2. The integrity of all cloned genes was confirmed by DNA sequencing.

### Proteins

GST, GST-Orf, MBP, MBP-Orf, SSB, SSB113 and SSBΔC10 proteins were purified as described [13]. Purified SSBΔ115-144 protein [27] was kindly provided by Robert Lloyd, University of Nottingham. MBP-YbcN was purified from 1 l of DH5*α* or BL21 (DE3) Rosetta carrying pLB101 grown in LB broth supplemented with 10 mM glucose and ampicillin at 150 µg/ml and 37 µg/ml chloramphenicol as required. Expression was induced with 0.5 mM IPTG when the culture reached an A_650nm_ of 0.5 and incubation continued for 3 hours at 37°C. Harvested cells were resuspended in column buffer (20 mM Tris-HCl pH7.4, 200 mM NaCl, 1 mM EDTA) and lysed by sonication. The cleared lysate was applied to a 4 ml amylose column (New England Biolabs) in column buffer and bound proteins eluted in the presence of 10 mM maltose. Fractions containing MBP-YbcN were collected and dialyzed in 20 mM Tris-HCl pH 8.0, 1 mM EDTA, 0.5 mM DTT, 200 mM KCl, 50% (v/v) glycerol. Aliquots were stored at -80°C and a total of 70 mg of highly pure MBP-YbcN was recovered at a concentration of 17.5 mg/ml. Additional stocks of MBP-YbcN were purified by both amylose and heparin agarose chromatography, with the majority of protein eluting from the latter matrix at 0.45 M NaCl.

GST-YbcN was purified from 6 l of BL21 Codon Plus cells carrying pLB108. Cells were grown at 25°C in LB broth containing ampicillin at 150 µg/ml and protein expression induced as before. Harvested cells were resuspended in PBS (140 mM NaCl, 2.7 mM KCl, 10 mM Na_2_HPO_4_, 1.8 mM KH_2_PO_4_ pH 7.3) and lysed in a cell disruptor at 15-25 psi. The supernatant was applied to a 4 ml glutathione sepharose 4B column (GE Healthcare) equilibrated in the same buffer. Fusion proteins were eluted in 50 mM Tris-HCl, 10 mM reduced glutathione pH 8.0 and further purified by passage through heparin agarose and Q sepharose chromatography, collecting the flow-though at each stage. A total of 32 mg GST-YbcN (16.2 mg/ml) was recovered.

MBP-Orf mutant proteins were purified from 500 ml of BL21-AI carrying the appropriate pMALc2 construct as described [13]. MBP-Orf Q45A, W50A, R103E and V106E were further purified by heparin-agarose chromatography after the amylose affinity purification step.

Protein concentrations were determined in a NanoDrop 2000 (Thermo Scientific) micro-volume spectrophotometer and by a modified Bradford assay (BioRad) using bovine serum albumin as a standard; amounts of Orf proteins are expressed as moles of dimer. Restriction endonucleases, T4 DNA ligase, T4 polynucleotide kinase and Pfx DNA polymerase were obtained from Invitrogen.

### DNA substrates

Oligonucleotides used in this study are listed in Table S4. The 50 nt ssDNA substrate (SS_50_) consisted of o-SS_50_, which was annealed to its complement (o-DS_50_) to give a 50 bp dsDNA duplex (DS_50_). The 60 nt ssDNA substrate (SS_60_) consisted of o-SS_60_, which was annealed to its complement (o-DS_60_) to give a fully complementary 60 bp dsDNA duplex (DS_60_). Centrally located mismatch (MM) or bubble (BB) structures of 1, 5, 13 and 20 nt (MM_G:G_, MM_T:G_, BB_5_, BB_13_ and BB_20_) were made by annealing o-SS_60_ with o-MM_G:G_, o-MM_T:G_, o-BB_5_, o-BB_13_ and o-BB_20_, respectively. Bent DNA substrates containing 1 (BT_1_), 2 (BT_2_) or 3 (BT_3_) adenine insertions were made by annealing o-SS_60_ with o-BT_1_, o-BT_2_ and o-BT_3_, respectively. In gel shift assays, o-SS_50_ or o-SS_60_ were labeled with [*γ* ^32^P] ATP at the 5′ end using T4 polynucleotide kinase. Labeled DNA was separated from unincorporated nucleotide using MicroBioSpin columns (BioRad). Annealed substrates were further purified by separation on 10% polyacrylamide gels in 90 mM Tris-borate, 2 mM EDTA. A 60 nt fluorescein 3**′**-end-labeled ssDNA equivalent in sequence to o-SS_60_ was used in fluorescence anisotropy experiments, alongside DS_60_, MM_T:G_ and BB_20_ substrates generated by annealing the fluorescein-labeled strand to appropriate partner oligonucleodides.

### DNA binding assays

Band shift assays (20 µl) with 50 and 60 nt ^32^P-labelled DNA substrates were conducted in 50 mM Tris-HCl pH 8.0, 5 mM EDTA, 1 mM dithiothreitol, 5% glycerol, 100 µg/ml BSA. Samples were incubated on ice for 15 min prior to separation on 4% PAGE in 6.7 mM Tris-HCl pH 8.0, 3.3 mM sodium acetate, 2 mM EDTA at 160 V. EDTA was replaced with 1 mM MgCl_2_ in both binding mixtures and gels to assess protein-DNA interactions in the presence of Mg^2+^ ions. Gels were dried on filter paper and analyzed by autoradiography and phosphorimaging. Data were analyzed using ImageQuant and ImageJ software.

DNA-protein interactions measured by fluorescence anisotropy utilized 3′-fluorescein-labeled derivatives of SS_60_, DS_60_, MM_T:G_ and BB_20_ in 100 mM Tris-HCl pH 7.0. Protein was titrated into 1 ml of 10 nM DNA in a 1 ml two-way quartz cuvette (10 mm path length). Changes in anisotropy were monitored on a modified Cary Eclipse Fluorescence Spectrophotometer (Agilent Technologies) fitted with polarizing filters with excitation at 494 nm and emission at 521 nm. Five replicates were taken with an averaging time of 20 sec. Data were analyzed using DynaFit 3 software to estimate the *K_D_* for MBP-YbcN, MBP-Orf wt and mutant proteins on SS_60_ and BB_20_.

### Size exclusion chromatography

The apparent molecular mass of proteins was estimated using an AKTA FPLC system with a 24 ml Superose 12 HR 10/30 column (GE Healthcare). Proteins (1 mg/ml) were applied to the gel filtration column in 20 mM Tris-HCl pH 8.0, 1 mM EDTA, 0.5 mM DTT, 150 mM KCl at a flow rate of 0.5 ml/min. Molecular mass standards (BioRad) contained thyroglobulin (670 kDa), *γ*-globulin (158 kDa), ovalbumin (44 kDa), myoglobin (17 kDa) and vitamin B12 (1.35 kDa).

### Far western blotting

Purified SSB wt and mutant proteins were separated on 15% SDS-PAGE and transferred to a PVDF membrane by electroblotting in 48 mM Tris, 39 mM glycine, 1.3 mM SDS. A prestained protein molecular weight standard (BioRad) served as a size marker. Blots were probed with YbcN or Orf fused to MBP or GST and interactions detected with monoclonal anti-MBP or anti-GST antibodies and mouse IgG peroxidase conjugate (Sigma). Chemiluminescence was observed by exposure to X-ray film following treatment with ECL reagents (GE Healthcare). Appropriate controls, either positive (appropriately tagged YbcN or Orf bound to the membrane) or negative (omitting tagged YbcN/Orf or including purified MBP or GST), were analyzed in parallel. Protein transfer to membranes was monitored by staining with 1% amido black (Sigma) in 10% acetic acid.

### ELISA

SSB wt and mutant proteins in PBS were applied to a flat bottom Immulon 2HB microtitre plate (Thermo Scientific) and incubated for 16 h at 37°C to allow binding. Wells were washed in PBS containing 0.3% Tween-20 (Sigma) and incubated for 1 hr at 37°C in this buffer containing 5% milk powder. After washing, GST-Orf or GST-YbcN proteins were added and incubated for 1 hr at 37°C. Wells were washed again prior to addition of HRP-anti-GST antibody (Sigma) at a 1∶1000 dilution. Tetramethylbenzidine (Sigma) was added and the intensity of the color reaction measured at A_620nm_ in an Anthos HTII plate reader.

### Static light scattering

Proteins at 0.2 mg/ml (50 µl) in 100 mM Tris-HCl pH 7 at 25°C were analyzed in a Zetasizer µV (Malvern Instruments Ltd). Thirteen measurements at 10 sec intervals were recorded and the data set repeated in triplicate for each sample.

### Circular dichroism (CD) spectroscopy

Proteins were dialyzed overnight in Slide-A-Lyzer Mini Dialysis Units (Pierce) in ultrapure water at 4°C. Dialyzed samples were centrifuged at 15 000 rpm for 30 minutes to remove any precipitated protein. Protein concentrations were determined by measurement of the absorbance at 280 nm. Far-UV CD spectra and the corresponding blanks were recorded on a Jasco J-810 Spectropolarimeter in a 1 ml cuvette (0.2 cm path length). Eight accumulations recorded at a rate of 10 nm/min, a pitch of 0.5 nm, a bandwidth of 1 nm and a response time of 2 s, were averaged. After subtraction of the appropriate blank, adaptive smoothing was carried out within the Jasco Spectra Analysis program (version 1.50, Jasco, Great Dunmow, UK). Smoothed data in the range 185-240 nm were analyzed for protein secondary structure using the CDSSTR, SELCON3, and CONTIN/LL programs in the CDPro package [54] with the SP37 reference set of soluble proteins [55]. Secondary structure content of known crystal structures was determined by DSSP [56] and accessed through the sequence retrieval system (SRS) at the European Bioinformatics Institute (http://srs.ebi.ac.uk).

## Supporting Information

Figure S1 Phylogram of representative Orf family proteins. The tree was obtained by the neighborhood joining method using *Staphylococcus aureus* phage 187 and *Listeria innocua* sequences lin1258 and lin1740 sequences as the outgroup. *S. typhimurium* phage ST64T and *E. coli* O157:H7 phage 933W were excluded from the phylogenetic tree because they carry incomplete *orf* genes. Only branches with significant values (>70%) are shown. Abbreviations are: *Eco*, *E. coli*; *Sso*, *Shigella sonnei*; *Plu*, *Photorhabdus luminescens*; *Sen*, *Salmonella enterica*; *Bbr*, *Bordetella bronchiseptica*; *Nar*, *Novosphingobium aromaticivorans*; *Mma*, *Magnetospirillum magnetotacticum*; *Psy*, *Pseudomonas syringae*; UPEC, Uropathogenic *E. coli*; *Sty*, *Salmonella enterica* serovar Typhimurium; *Sfl*, *Shigella flexneri*; *Ype*, *Yersinia pestis*; *Ahy*, *Aeromonas hydrophila*; *Hin*, *Haemophilus influenzae*; *Aac*, *Actinobacillus actinomycetemcomitans*; *Ngo*, *Neisseria gonorrhoeae*; *Bvi*, *Burkholderia vietnamiensis*; *Lin*, *Listeria innocua*; *Sau*, *Staphylococcus aureus*. Four groups of closely-related homologs are highlighted in color. The true phylogeny is difficult to ascertain due to the likelihood of gene transfer between phage genomes. Full designations of sources are given in Table S1.
(TIFF)

Figure S2 Structural similarity between *λ* Orf, DLP12 YbcN and ***φ***ETA Orf20 proteins. Structural models of *E. coli* DLP12 YbcN (residues 16-147), belonging to the PRK09741 domain, and *S. aureus* ***φ***ETA Orf20 (residues 26-130), a member of the DUF968 domain, were based on the *λ* Orf crystal structure (1PC6) using Phyre^2^. Only subunit A is shown, with the RAGNYA domain colored in yellow. Conserved and potential functionally-equivalent residues located close to the central channel of the dimer are highlighted.
(TIFF)

Figure S3 YbcN binding to single-stranded and bubble DNA. Comparison of MBP-YbcN binding to 10 nM fluorescein-labeled SS_60_ and BB_20_ as determined by fluorescence anisotropy. Data are the mean and standard deviation of two independent experiments.
(TIFF)

Figure S4 YbcN and Orf binding to mismatch and bent DNA. (**A**) Comparison of MBP-YbcN and MBP-Orf binding to 10 nM fluorescein-labeled MM_G:G_ and MM_T:G_ DNA as determined by fluorescence anisotropy. Data are the mean and standard deviation of two independent experiments. (**B**) Comparison of MBP-YbcN and MBP-Orf binding to bent DNA. Gel mobility shift assays contained 125 nM MBP-Orf (O) or MBP-YbcN (Y) proteins, 5 mM EDTA and 0.15 nM of ^32^P-labelled 60 nt (SS_60_) ssDNA (lanes a-c), 60 bp (DS_60_) dsDNA (lanes d-f), 1 nt (BT_1_) insertion (lanes g-i), 2 nt (BT_2_) insertion (lanes j-l) and 3 nt (BT_3_) insertion (lanes m-o).
(TIFF)

Figure S5 Analysis of DLP12 MBP-YbcN and GST-YbcN proteins. (**A**) CD analysis of YbcN proteins and binding to ssDNA. CD spectra (180–260 nm) were obtained for MBP, GST, MBP-YbcN and GST-YbcN proteins in ultrapure water at 20°C. Gel shift assays contained 0.3 nM ^32^P-labelled ssDNA (SS_50_), 5 mM EDTA and 62.5, 125 and 250 nM MBP-Orf (lanes b-d) and 250, 500 and 1000 nM MBP-YbcN (lanes f-h) and GST-YbcN (lanes j-l). (**B**) Size-exclusion chromatography of DLP12 YbcN. MBP-YbcN and GST-YbcN proteins (1 mg/ml) were applied to a 24 ml Superose 6HR 10/30 column in 20 mM Tris-HCl pH8, 1 mM EDTA, 0.5 mM DTT, 250 mM KCl. The predicted molecular weights for each protein monomer are 60.4 kDa for MBP-YbcN and 44.6 kDa for GST-YbcN. Oligomeric states are depicted with a circle representing a single subunit and placed adjacent to the corresponding peak. Boiled (+) and unboiled (–) samples of each purified protein were separated on 12.5% SDS-PAGE and stained with Coomassie blue.
(TIFF)

Figure S6 MBP-Orf mutant protein binding to SSB in far western assays. SSB protein (5 µg) separated on 15% SDS-PAGE was blotted and probed with 30 µg MBP-Orf mutant proteins. MBP-Orf-SSB interactions were detected with antibodies specific for the MBP domain.
(TIFF)

Table S1 Representative Orf family proteins.
(PDF)

Table S2 Secondary structure predictions for YbcN and Orf mutant proteins from CD data.
(PDF)

Table S3 Static light scattering analysis of MBP-Orf mutants.
(PDF)

Table S4 Oligonucleotides used to generate DNA substrates.
(PDF)
