# Supplementary figures and images for: Phage Orf Family Recombinases: Conservation of Activities and Involvement of the Central Channel in DNA Binding
Source: PLoS One. 2014 Aug 1;9(8):e102454. doi: 10.1371/journal.pone.0102454 (PMC4118853; doi:10.1371/journal.pone.0102454)

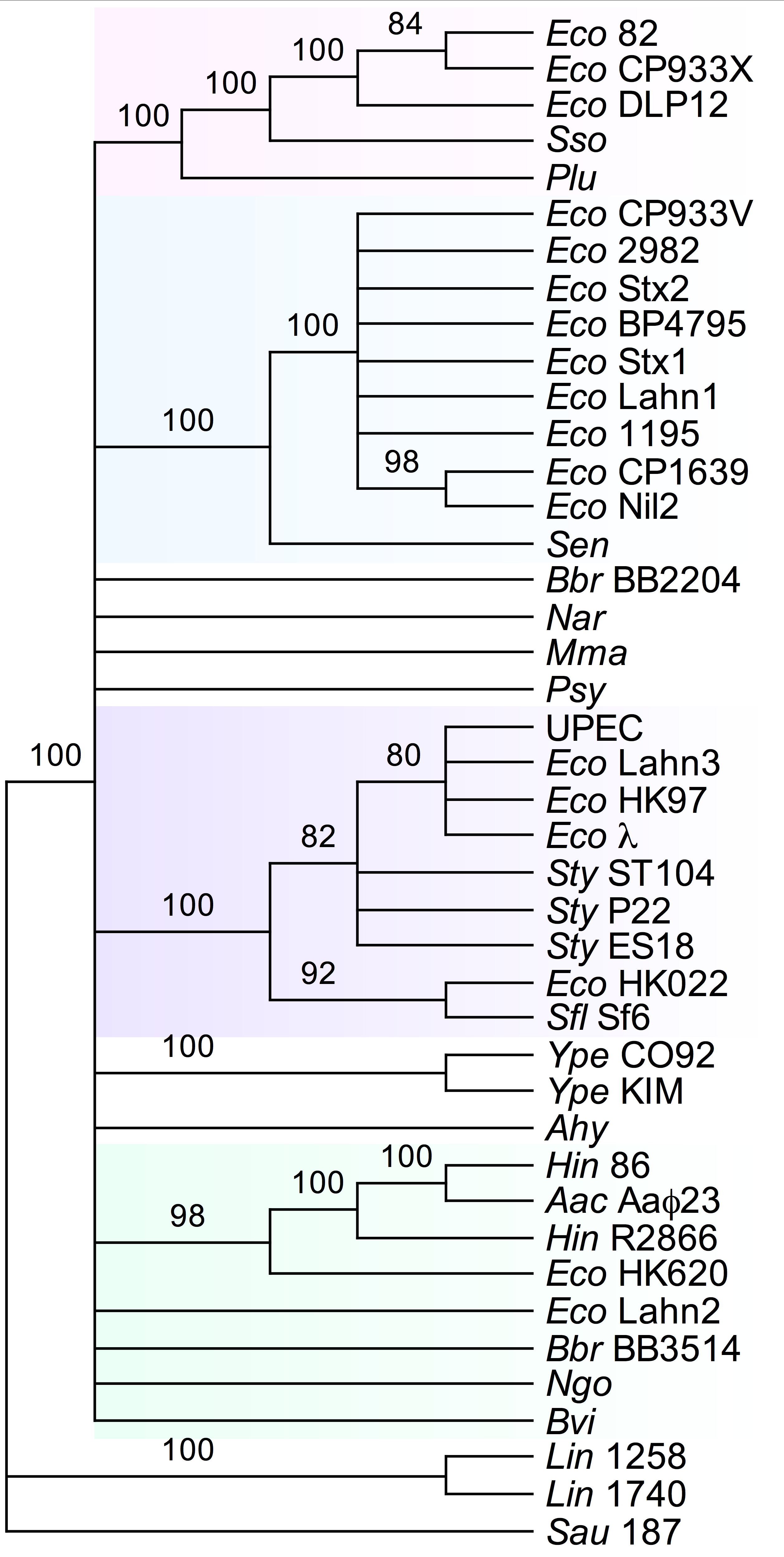

Supplement: Figure S1 — Phylogram of representative Orf family proteins. The tree was obtained by the neighborhood joining method using Staphylococcus aureus phage 187 and Listeria innocua sequences lin1258 and lin1740 sequences as the outgroup. S. typhimurium phage ST64T and E. coli O157:H7 phage 933W were excluded from the phylogenetic tree because they carry incomplete orf genes. Only branches with significant values (>70%) are shown. Abbreviations are: Eco, E. coli; Sso, Shigella sonnei; Plu, Photorhabdus luminescens; Sen, Salmonella enterica; Bbr, Bordetella bronchiseptica; Nar, Novosphingobium aromaticivorans; Mma, Magnetospirillum magnetotacticum; Psy, Pseudomonas syringae; UPEC, Uropathogenic E. coli; Sty, Salmonella enterica serovar Typhimurium; Sfl, Shigella flexneri; Ype, Yersinia pestis; Ahy, Aeromonas hydrophila; Hin, Haemophilus influenzae; Aac, Actinobacillus actinomycetemcomitans; Ngo, Neisseria gonorrhoeae; Bvi, Burkholderia vietnamiensis; Lin, Listeria innocua; Sau, Staphylococcus aureus. Four groups of closely-related homologs are highlighted in color. The true phylogeny is difficult to ascertain due to the likelihood of gene transfer between phage genomes. Full designations of sources are given in Table S1. (TIFF) [file pone.0102454.s001.tiff]

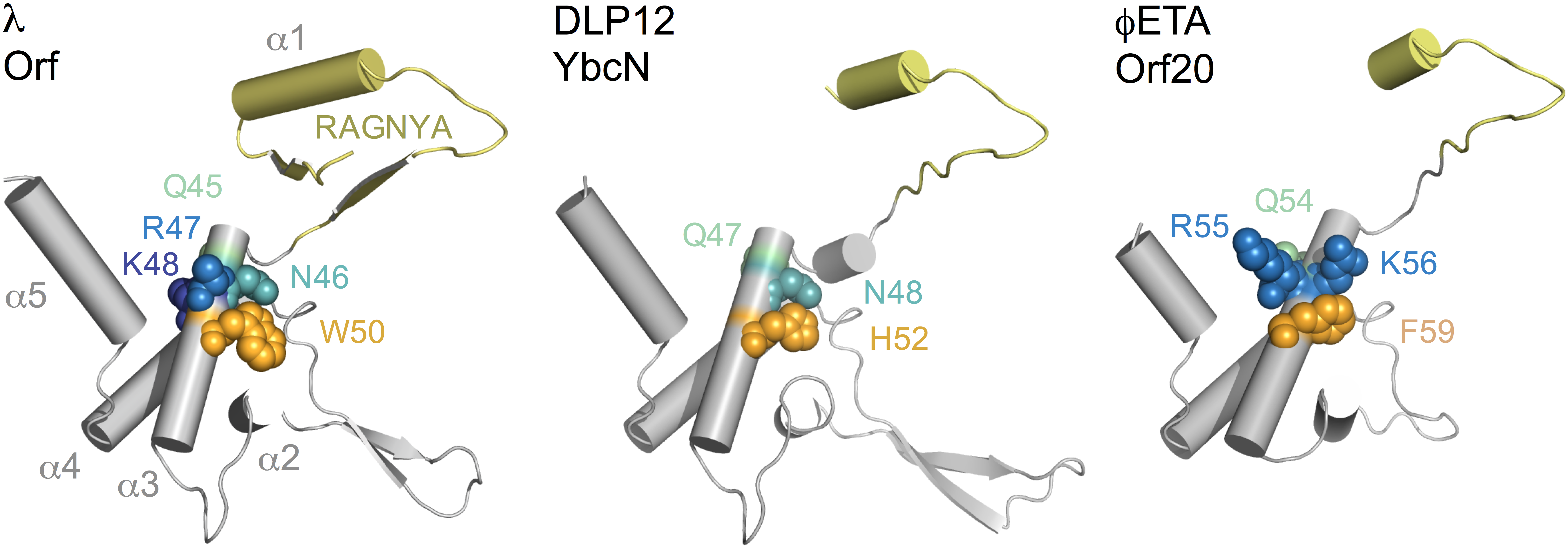

Supplement: Figure S2 — Structural similarity between λ Orf, DLP12 YbcN and φETA Orf20 proteins. Structural models of E. coli DLP12 YbcN (residues 16-147), belonging to the PRK09741 domain, and S. aureus φETA Orf20 (residues 26-130), a member of the DUF968 domain, were based on the λ Orf crystal structure (1PC6) using Phyre2. Only subunit A is shown, with the RAGNYA domain colored in yellow. Conserved and potential functionally-equivalent residues located close to the central channel of the dimer are highlighted. (TIFF) [file pone.0102454.s002.tiff]

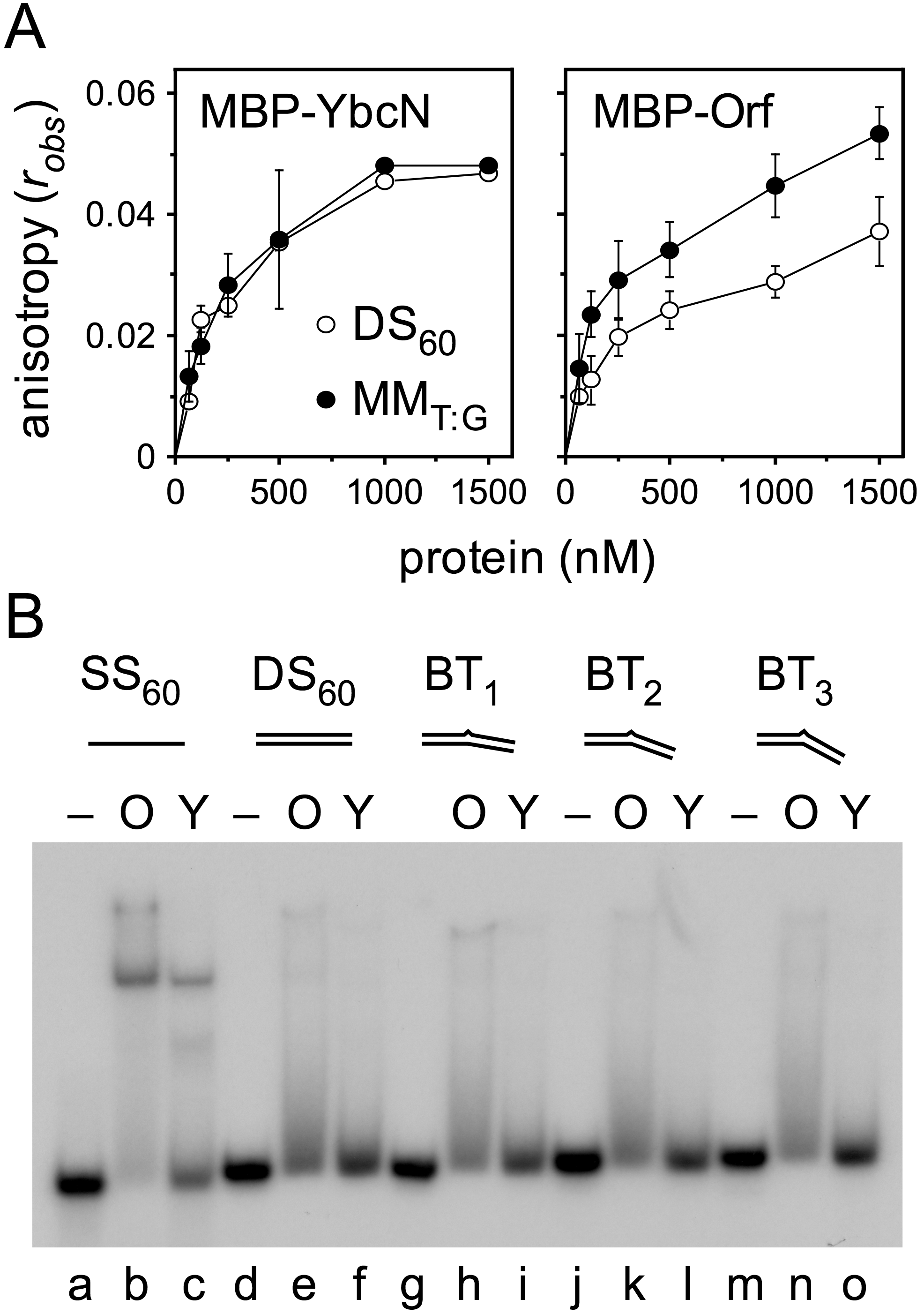

Supplement: Figure S4 — YbcN and Orf binding to mismatch and bent DNA. (A) Comparison of MBP-YbcN and MBP-Orf binding to 10 nM fluorescein-labeled MMG:G and MMT:G DNA as determined by fluorescence anisotropy. Data are the mean and standard deviation of two independent experiments. (B) Comparison of MBP-YbcN and MBP-Orf binding to bent DNA. Gel mobility shift assays contained 125 nM MBP-Orf (O) or MBP-YbcN (Y) proteins, 5 mM EDTA and 0.15 nM of 32P-labelled 60 nt (SS60) ssDNA (lanes a-c), 60 bp (DS60) dsDNA (lanes d-f), 1 nt (BT1) insertion (lanes g-i), 2 nt (BT2) insertion (lanes j-l) and 3 nt (BT3) insertion (lanes m-o). (TIFF) [file pone.0102454.s004.tiff]

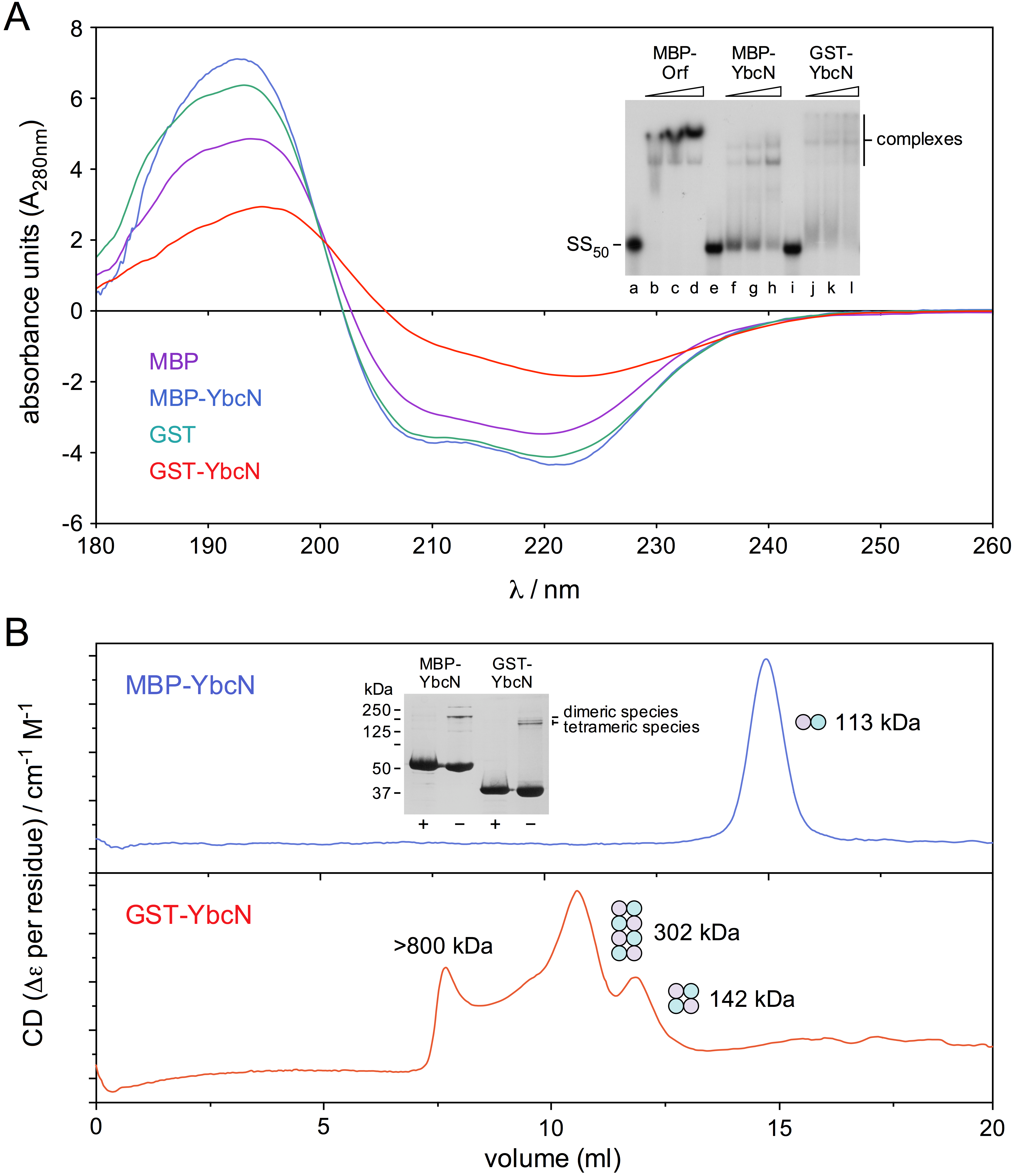

Supplement: Figure S5 — Analysis of DLP12 MBP-YbcN and GST-YbcN proteins. (A) CD analysis of YbcN proteins and binding to ssDNA. CD spectra (180–260 nm) were obtained for MBP, GST, MBP-YbcN and GST-YbcN proteins in ultrapure water at 20°C. Gel shift assays contained 0.3 nM 32P-labelled ssDNA (SS50), 5 mM EDTA and 62.5, 125 and 250 nM MBP-Orf (lanes b-d) and 250, 500 and 1000 nM MBP-YbcN (lanes f-h) and GST-YbcN (lanes j-l). (B) Size-exclusion chromatography of DLP12 YbcN. MBP-YbcN and GST-YbcN proteins (1 mg/ml) were applied to a 24 ml Superose 6HR 10/30 column in 20 mM Tris-HCl pH8, 1 mM EDTA, 0.5 mM DTT, 250 mM KCl. The predicted molecular weights for each protein monomer are 60.4 kDa for MBP-YbcN and 44.6 kDa for GST-YbcN. Oligomeric states are depicted with a circle representing a single subunit and placed adjacent to the corresponding peak. Boiled (+) and unboiled (–) samples of each purified protein were separated on 12.5% SDS-PAGE and stained with Coomassie blue. (TIFF) [file pone.0102454.s005.tiff]

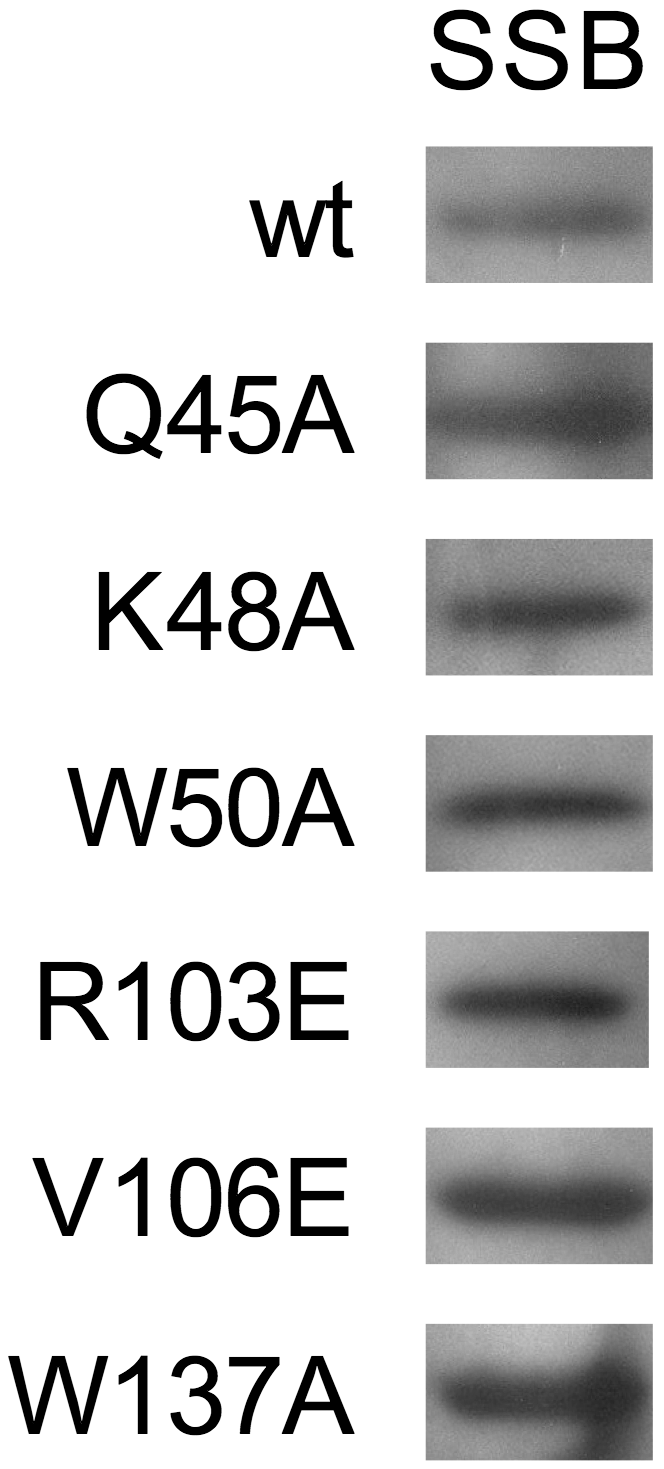

Supplement: Figure S6 — MBP-Orf mutant protein binding to SSB in far western assays. SSB protein (5 µg) separated on 15% SDS-PAGE was blotted and probed with 30 µg MBP-Orf mutant proteins. MBP-Orf-SSB interactions were detected with antibodies specific for the MBP domain. (TIFF) [file pone.0102454.s006.tiff]
