# Supplementary material for: Phage Orf Family Recombinases: Conservation of Activities and Involvement of the Central Channel in DNA Binding
Source: PLoS One. 2014 Aug 1;9(8):e102454. doi: 10.1371/journal.pone.0102454 (PMC4118853; doi:10.1371/journal.pone.0102454)
Supplement: Table S1 — Representative Orf family proteins. (PDF) [file pone.0102454.s007.pdf]

Table S1. Representative Orf family proteins

Accession	Length	Source	Identity (%)
AAA96587	146	Bacteriophage λ	100
AAN80023	146	*Escherichia coli* CFT073 (Uropathogenic *E. coli*)	99
CAC95061	146	*Escherichia coli* O84:H^{-} phage Lahn3	98
AAF31136	146	Enterobacteria phage HK97	98
AAW70535	145	*Salmonella enterica* Typhimurium phage ES18	97
BAD15193	145	*Salmonella enterica* Typhimurium phage ST104	96
NP597901	146	Enterobacteria phage HK022	95
AAF75030	145	Enterobacteria phage P22	95
AAQ12239	146	*Shigella flexneri* phage Sf6	94
CAL20725	142	*Yersinia pestis* CO92	55
AAM85783	148	*Yersinia pestis* KIM	54
AAS46709	146	*Aeromonas hydrophila*	52
ZP00208985	138	*Magnetospirillum magnetotacticum* MS-1	48
AAL15506	101	*Salmonella typhimurium* phage ST64T (truncated)	44
CAB39290	111	*Escherichia coli* O157:H7 phage 933W (truncated)	44
CAJ26398	138	*Escherichia coli* O84:H^{-} phage Lahn2	44
AAY37846	132	*Pseudomonas syringae* B728a	43
BAA84314	181	*Escherichia coli* O157:H7 Stx2-converting phage VT2-Sa	42
BAC77948	181	*Escherichia coli* O157:H7 Stx1-converting phage	42
CAD88833	181	*Escherichia coli* O84:H^{-} phage BP-4795	42
BAB34618	148	*Escherichia coli* O157:H7 (ECs1195)	42
BAB36405	148	*Escherichia coli* O157:H7 (ECs2982)	42
AAG57234	148	*Escherichia coli* O157:H7 EDL933 prophage CP-933V	42
CAC95070	148	*Escherichia coli* O84:H^{-} phage Lahn1	42
CAC83141	181	*Escherichia coli* 1639/77 phage CP-1639	41
AAV78288	148	*Salmonella enterica* Paratyphi A strain ATCC9150	41
CAC95094	181	*Escherichia coli* O157:H7 phage Nil2	41
AAK28875	136	*Escherichia coli* H phage HK620	37
CAE32701	135	*Bordetella bronchiseptica* RB50 (BB2204)	34
ZP00156617	138	*Haemophilus influenzae* R2866	34
AAX88515	138	*Haemophilus influenzae* 86-028NP	33
CAD90796	165	*Actinobacillus actinomycetemcomitans* phage Aaf23	32
ABD27460	124	*Novosphingobium aromaticivorans* DSM 12444	30
AAW89770	127	*Neisseria gonorrhoeae* FA 1090	29
EAM26711	136	*Burkholderia vietnamiensis* G4	28
CAE34008	151	*Bordetella bronchiseptica* RB50 (BB3514)	25
CAC96971	186	*Listeria innocua* Clip11262 (lin1740)	18
CAC96489	184	*Listeria innocua* Clip11262 (lin1258)	18
AAZ89087	151	*Shigella sonnei* Ss046	17
CAE15288	147	*Photorhabdus luminescens* TTO1	16
AAG55969	151	*Escherichia coli* O157:H7 EDL933 (phage CP-933X)	16
CAA63327	151	*Escherichia coli* phage 82	16

S. enterica serovar Typhimurium phage ST64T and *E. coli* O157:H7 phage 933W were excluded from the phylogenetic tree (Figure S1) because they carry incomplete *orf* genes. Similarly, *L. innocua* Clip11262 (lin1740) was omitted because it differs from lin1258 at only eight positions.
