# Supplementary material for: Phage Orf Family Recombinases: Conservation of Activities and Involvement of the Central Channel in DNA Binding
Source: PLoS One. 2014 Aug 1;9(8):e102454. doi: 10.1371/journal.pone.0102454 (PMC4118853; doi:10.1371/journal.pone.0102454)
Supplement: Table S2 — Secondary structure predictions for YbcN and Orf mutant proteins from CD data. (PDF) [file pone.0102454.s008.pdf]

Table S2. Secondary structure predictions for YbcN and Orf mutant proteins from CD data.

	α-helix	β-sheet	Turn	Coil
MBP[a]	45	28	12	15
GST[a]	50	7	13	29
MBP-Orf[a,b]	46	27	11	16
MBP	31	21	19	31
MBP-YbcN	42	12	16	31
GST	20	29	21	30
GST-YbcN	17	33	21	29
MBP-Orf	38	16	15	31
Q45A	36	13	21	31
K48A	49	9	12	30
W50A	40	10	20	31
R103E	39	14	16	30
V106E	39	17	15	29
W137A	42	15	15	29

Comparison of MBP-Orf secondary structures as determined by analysis of the known crystal structures[a] of MBP, GST and Orf (PDB codes: 1fqa, 1m99 and 1pc6, respectively) and deconvolution of the CD data. In the case of MBP-Orf[b] the statistics for 1fqa and 1pc6 were combined. CD spectra for each protein were analyzed using the CDSSTR, ContinLL and Selcon3 programs from the CDPro suite. The ContinLL provided the best fit and percentage data for this method are shown.
