# Supplementary material for: Phage Orf Family Recombinases: Conservation of Activities and Involvement of the Central Channel in DNA Binding
Source: PLoS One. 2014 Aug 1;9(8):e102454. doi: 10.1371/journal.pone.0102454 (PMC4118853; doi:10.1371/journal.pone.0102454)
Supplement: Table S3 — Static light scattering analysis of MBP-Orf mutants. (PDF) [file pone.0102454.s009.pdf]

Table S3. Static light scattering analysis of MBP-Orf mutants.

	Z-average	Pd Index	% Pd	r (nM)	kDa
MBP	22.68 ±12.18	0.288	26.7	17.88 ±4.770	2290 ±764.8
MBP-Orf	43.88 ±0.747	0.548	12.2	6.11 ±0.747	212.3 ±79.8
Q45A	20.30 ±18.86	0.863	12.0	5.80 ±0.694	190.6 ±24.6
K48A	45.28 ±35.78	0.623	10.6	5.48 ±0.579	171.0 ±52.0
W50A	17.12 ±17.12	1.000	16.1	5.82 ±0.937	190.6 ±45.3
R103E	6.21 ±4.219	0.462	15.2	5.24 ±0.798	153.5 ±34.3
V106E	72.60 ±48.52	0.447	55.8	114.90 ±64.15	139000 ±124000
W137A	58.53 ±41.22	0.496	7.0	5.91 ±0.415	212.0 ±87.4

MBP and MBP-Orf proteins at 0.2 mg/ml in 100 mM Tris-HCl pH7 at 25°C were analyzed in a Zetasizer µV. The polydispersity (Pd) index for each sample and the percentage for the predominant MBP-Orf peak are shown. Standard deviations of the duplicate data sets are shown.
