# Supplementary material for: Phage Orf Family Recombinases: Conservation of Activities and Involvement of the Central Channel in DNA Binding
Source: PLoS One. 2014 Aug 1;9(8):e102454. doi: 10.1371/journal.pone.0102454 (PMC4118853; doi:10.1371/journal.pone.0102454)
Supplement: Table S4 — Oligonucleotides used to generate DNA substrates. (PDF) [file pone.0102454.s010.pdf]

Table S4. Oligonucleotides used to generate DNA substrates.

Oligo	Nucleotide sequence (5'-3')
o-SS$_{50}$	GGCGACGTGATCACCAGATGATTGCTAGGCATGCTTTCCGCAAGAGAAGC
o-DS$_{50}$	GCTTCTCTTGCGGAAAGCATGCCTAGCAATCATCTGGTGATCACGTCGCC
o-SS$_{60}$	TTTGGTCTAACTTTACCGCTACTAAATGCCGCGGATTGGTTTCGCTGAATCAGGTTATTA
o-DS$_{60}$	TAATAACCTGATTCAGCGAAACCAATCCGCGGCATTTAGTAGCGGTAAAGTTAGACCAAA
o-MM$_{G:G}$	TAATAACCTGATTCAGCGAAACCAATCGGCGGCATTTAGTAGCGGTAAAGTTAGACCAAA
o-MM$_{T:G}$	TAATAACCTGATTCAGCGAAACCAATCTGCGGCATTTAGTAGCGGTAAAGTTAGACCAAA
o-BB$_5$	TAATAACCTGATTCAGCGAAACCAATCGCAACCATTTAGTAGCGGTAAAGTTAGACCAAA
o-BB$_{13}$	TAATAACCTGATTCAGCGAAACATTGTAGGTAAGCTTAGTAGCGGTAAAGTTAGACCAAA
o-BB$_{20}$	TAATAACCTGATTCAGCGAATGACCGATAACGTCCACTTGAGCGGTAAAGTTAGACCAAA
o-BT$_1$	TAATAACCTGATTCAGCGAAACCAATCCAGCGGCATTTAGTAGCGGTAAAGTTAGACCAAA
o-BT$_2$	TAATAACCTGATTCAGCGAAACCAATCCAAGCGGCATTTAGTAGCGGTAAAGTTAGACCAAA
o-BT$_3$	TAATAACCTGATTCAGCGAAACCAATCCAAAGCGGCATTTAGTAGCGGTAAAGTTAGACCAAA

Nucleotides underlined in o-MM$_{G:G}$, o-MM$_{T:G}$, o-BB$_5$, o-BB$_{13}$ and o-BB$_{20}$ highlight sequences that differ from o-SS$_{60}$ and generate mismatch or bubble structures when annealed. Similarly, underlined nucleotides in o-BT$_1$ to o-BT$_3$ indicate sequences that generate insertions when annealed to o-SS$_{60}$.
